# Meta-analysis of perioperative amiodarone for prevention of postoperative atrial fibrillation (POAF) in cardiac surgery patients: update and reevaluation of timing, route, and dosage

**DOI:** 10.1186/s12872-026-05813-w

**Published:** 2026-05-07

**Authors:** Zemeng Li, Shilin Wang, Lili An, Chen Chen, Guitao Zhang, Liang Chen, Juan Du

**Affiliations:** 1 Department of Surgical Intensive Care Unit, Fuwai Hospital, National Center for Cardiovascular Diseases, Chinese Academy of Medical Science and Peking Union Medical College, Beijing, 100037 China; 2 Department of Cardiovascular Surgery, Fuwai Hospital, National Center for Cardiovascular Diseases, Chinese Academy of Medical Sciences and Peking Union Medical College, Beijing, 100037 China; 3 Department of Neurology, National Clinical Research Center for Cardiovascular Diseases, Fuwai Hospital, National Center for Cardiovascular Diseases, Chinese Academy of Medical Sciences and Peking Union Medical College, Beijing, 100037 China

**Keywords:** Postoperative atrial fibrillation, Amiodarone, Cardiac surgery, Coronary artery bypass grafting, Meta-analysis

## Abstract

**Background:**

Postoperative atrial fibrillation (POAF) is a common complication following cardiac surgery, and perioperative amiodarone is recommended for POAF prophylaxis, while the optimal timing, route, and dosage remain unclear. The purpose of this study is to evaluate the efficacy of perioperative amiodarone for the prevention of POAF in patients undergoing cardiac surgery and to reevaluate the impact of its timing, route, and dosage.

**Methods:**

Data were collected through searching PubMed, Embase, and the Cochrane Library from inception until September 30, 2025, for randomized controlled trials (RCTs). Data were pooled using a random-effects model.

**Results:**

Forty RCTs involving 6,166 patients were included. Amiodarone was associated with a substantial reduction in the incidence of POAF (odds ratio [OR] 0.39, 95% confidence interval [CI] 0.31 to 0.49, *P* < 0.00001, *I*^2^ = 57%). The preventive efficacy may be primarily influenced by the combination of administration timing and route, rather than by the cumulative dose alone. Notably, a significant dose–response relationship was observed within the preoperative through postoperative oral strategy. Statistically significant differences were found in length of hospital stay (mean difference -1.33 days, *P* < 0.0001) and cerebrovascular accident (OR = 0.59, *P* = 0.04), and an increased risk of bradycardia (OR = 2.33, *P* < 0.00001). No statistically significant differences were found in mortality, heart block, or hypotension.

**Conclusions:**

Prophylactic perioperative amiodarone may be associated with a reduced incidence of POAF, consistent with current guideline recommendations, and the timing and route of administration appear to play a more important role than the dose alone. While an increased risk of bradycardia was observed, no clear association with major adverse outcomes was identified. These results should be interpreted cautiously and may help optimize prophylactic strategies in appropriate clinical contexts.

**Supplementary Information:**

The online version contains supplementary material available at 10.1186/s12872-026-05813-w.

## Introduction

Postoperative atrial fibrillation (POAF) is a common postoperative complication after coronary artery bypass grafting (CABG), with an incidence of approximately 20%, and the second to fourth day postoperatively is the peak period of occurrence [1]. According to previous research, atrial fibrillation (AF) is the primary reason for readmission within 30 days and 90 days after CABG, accounting for a quarter of total admissions [2]. For those who went through POAF during hospitalization, the cumulative incidence of AF in the first year is 10.0%, and by the fourteenth year, this number grows to 33.1% [3]. POAF has always been considered to be related to in-hospital adverse events, such as stroke, heart failure (HF), acute kidney injury (AKI), and prolonged intensive care unit (ICU) length of stay (LOS) (ILOS) and hospital LOS (HLOS) [4–6]. POAF also correlates with a higher risk of HF hospitalization, stroke, cardiovascular mortality, or all-cause mortality (ACM) [7, 8].

The management of POAF after cardiac surgery is distinct from that of persistent AF, which focuses on rhythm control with multiple drugs or procedures, as it prioritizes prevention [9–11]. Consequently, numerous pharmacological and surgical preventive approaches have been investigated [1]. Amiodarone is a classic antiarrhythmic drug that has been applied to the treatment of AF [9, 10, 12]. There are some AF management guidelines that recommend perioperative use of amiodarone to prevent POAF [9, 10, 13–15]. The 2024 ESC/SACTS guideline [10] and the 2023 ACC/AHA guideline [9] both recommend the use of amiodarone in the perioperative period of cardiac surgery for the prevention of POAF, with levels of recommendation and evidence are I/A and 2a/B-R, respectively. Preceding clinical trials have shown that amiodarone can be used for the prevention of POAF; its safety and effectiveness are guaranteed. However, the timing, route, and dosage of amiodarone vary from trial to trial. So, we want to explore what kind of timing, route, and dosage are more suitable for cardiac surgery patients and perform this meta-analysis in order to update and reevaluate amiodarone usage.

## Methods

### Study protocol and registration

Following the Preferred Reporting Items for Systematic Reviews and Meta-Analysis Quality of Reporting of Meta-Analysis (PRISMA) Guidelines, we conducted a meta-analysis [16]. This work has been registered on the International Prospective Register of Systematic Reviews (PROSPERO: CRD420251080182).

### Eligibility criteria of studies

We included only English-language randomized controlled trials (RCTs) of patients undergoing CABG or other cardiac surgeries who were randomly allocated to an intervention group (amiodarone) or a control group (placebo or no treatment) to prevent POAF.

We excluded studies in non-English languages, observational studies, and animal models, and we also excluded review articles, conference abstracts, editorial comments, letters, and case reports.

### Eligibility criteria of participants

Patients included in the studies need to be adults (≥ 18 years old) undergoing CABG, valvular surgery, a combination of the above two, or other types of cardiac surgeries. There was no restriction on the use of cardiopulmonary bypass (CPB). Patients with heart transplantation, thoracic surgeries, or other organ surgeries were excluded.

In addition, patients should have no history of AF, so studies with the maze procedure or catheter ablation were excluded. Besides, studies using amiodarone for cardioversion after the occurrence of POAF were also excluded.

### Interventions and controls

To assess the prophylactic effect of amiodarone versus placebo or no treatment on POAF, there is no restriction on the timing, route, or dosage of amiodarone administration.

The intervention group was given amiodarone. The administration timing of prophylactic amiodarone was usually divided into preoperative, intraoperative, postoperative, or any combination of the above time periods, and the administration route was usually divided into intravenous, oral, or a combination of the two, or intracardiac poly-based hydrogel materials.

The control group was given a placebo or no treatment. The placebo given to the control group is usually a matching placebo as an oral tablet, a dextrose aqueous solution, or normal saline as an injectable preparation.

### Outcome measures

#### Primary outcomes measures

The primary outcome we were interested in was the incidence of POAF, and the specific definition of POAF depends on the trials.

However, several early RCTs evaluating prophylactic amiodarone after cardiac surgery reported atrial flutter (AFL) or supraventricular tachyarrhythmia (SVT) as a composite endpoint without clearly distinguishing AF from other supraventricular arrhythmias. Therefore, when AF-specific data were unavailable, AFL or SVT endpoints were extracted as a proxy outcome for inclusion in the primary analysis. Sensitivity analyses were further performed, restricting the outcome definition to studies that explicitly reported POAF.

Considering that the primary analysis of our study was the incidence of POAF, articles on prophylactic administration of amiodarone that did not include POAF as a primary or secondary endpoint would be excluded.

#### Secondary outcomes measures

The secondary outcomes we want to evaluate were cerebrovascular accident (CVA), ACM, cardiovascular mortality (CVM), HLOS, ILOS, bradycardia, heart block, and hypotension. The specific definitions depend on the trials.

### Search strategy and selection

#### Databases and search terms

A comprehensive literature search on PubMed, Embase, and the Cochrane Library from inception until 30 September 2025 was conducted based on the following terms: “postoperative,” “atrial fibrillation,” “coronary artery bypass grafting,” and “amiodarone.” Detailed search strategies of each database are available in Supplementary Appendix 1.

#### Screening stage

All relevant articles would be imported into EndNote, and duplicates would be removed afterwards. Two researchers (Li Z and Wang S) independently screened the titles and abstracts of the remaining articles to remove apparently ineligible ones. For articles that may meet the eligibility criteria, the full text will be obtained for further evaluation.

#### Evaluating stage

If the article was not available in full text, or its main text was written in a non-English language, or for other reasons it cannot be eligible, it would be excluded. Two researchers (Li Z and Wang S) independently evaluated the full-text articles to determine the final ones. If there is a disagreement, it would be discussed with and decided by a senior researcher (Chen C).

### Data extraction and management

Two researchers (Li Z and Wang S) independently extracted data to a specified data extraction sheet, consisting of the following information: (1) eligible studies’ characteristics (author, affiliation, publication date, journal, title, study design, sample sizes, and groups), (2) eligible studies’ participant characteristics (age, gender, surgery type, left ventricular ejection fraction [LVEF]), (3) intervention details (start and end time, route, and dosage of amiodarone), (4) outcome measures (definition and incidence of POAF; CVA, ACM, CVM, ILOS, and HLOS), and (5) adverse effects of intervention (bradycardia, heart block, and hypotension). During the data extraction procedure, disagreements were resolved by three researchers (Li Z, Wang S, and Chen C).

If there were one intervention group receiving amiodarone and another intervention group receiving other precautions, respectively, in the same study, only the amiodarone intervention group data would be enrolled. For studies comprising two amiodarone intervention arms and a single shared control group, each intervention arm was treated as an independent trial for inclusion. To avoid double-counting of the shared control group, we divided the events of the control group between the two comparisons (labeled as ‘1’ and ‘2’ in the analysis). This approach treats each comparison as an independent study in the analysis while preserving the integrity of the original data. To ensure the sum of the split events equaled the original control group total, we applied a complementary rounding strategy based on the comparison label. Specifically, for comparisons labeled as ‘1’, we applied rounding up to the calculated split event count; for comparisons labeled as ‘2’, we applied rounding down. For example, if a control group originally had 3 events, we assigned 2 events (rounded up) to comparison ‘1’ and 1 event (rounded down) to comparison ‘2’, thereby maintaining the original total of 3 events.

The data collection was restricted to published studies. Unpublished data were not sought.

### Risk of bias assessment

Based on the Cochrane Handbook for Systematic Reviews of Interventions criteria and technique, two researchers (Li Z and An L) assessed the risk of bias of included studies using the Cochrane risk of bias 2 (ROB-2) tool independently [17], involving those items: (1) bias arising from the randomization process, (2) bias due to deviations from intended interventions, (3) bias due to missing outcome data, (4) bias in measurement of the outcome, (5) bias in selection of the reported result, and (6) overall bias. Assessment of bias risk yielded the following categorizations for each study: “low risk,” “high risk,” or “some concerns.” Risk of bias assessment was based on the aim of assessing the effect of intervention allocation ("intention-to-treat" effect) and on the primary study endpoint. Any disagreement will be eliminated through consulting the third researcher (Chen C). Publication bias was assessed using Egger's test, Harbord's test, and Peter's test and visualized with a contour-enhanced funnel plot.

### Data analysis and synthesis

Data analysis was performed using RevMan and R software. Dichotomous outcomes were combined into odds ratios (OR) using the Mantel–Haenszel random-effects model, with between-study variance estimated using the DerSimonian and Laird (DL) method. Continuous outcomes were combined into a mean difference (MD) presented with 95% confidence intervals (CI) using an inverse variance random-effects model, with between-study variance estimated using the DL method. A *P*-value < 0.05 was considered to indicate a statistically significant difference.

For random-effects meta-analyses conducted in RevMan, we estimated the CI of the summary effect using either the Hartung-Knapp-Sidik-Jonkman (HKSJ) method or the Wald-type (WT) method. Referring to the principles outlined in the Cochrane Handbook (Sects. 10.10.4.4 and 10.10.4.5), our decision rules, adapted for RevMan's analytical framework, were as follows. For the primary outcome and its associated sensitivity analyses or secondary outcomes, we used the HKSJ method for these analyses when the number of included studies was more than 3 and the estimated Tau^2^ was greater than 0; otherwise, the WT method was used. For analyses involving subgroups, due to the limitation of RevMan, which applies a single CI method uniformly across all subgroups, we adopted a conservative approach and applied the HKSJ method to the entire subgroup analysis if any of the individual subgroups both contained more than 3 studies and had an estimated Tau^2^ greater than 0; otherwise, the WT method was used.

### Heterogeneity assessment

The presence and extent of statistical heterogeneity will be assessed using the Cochran's Q Chi^2^ test, the I^2^ statistic, and the Tau^2^ statistic. The Cochran's Q Chi^2^ test, with a significance level of *P* < 0.10, was used to indicate statistical significance of heterogeneity. The I^2^ statistic, which quantifies the proportion of total variation across studies that is due to heterogeneity rather than chance, and ≥ 50% was considered to represent substantial heterogeneity. The Tau^2^ statistic estimates the variance of the true effect sizes between studies.

### Subgroup analysis

When substantial heterogeneity was detected (I^2^ > 50%), pre-specified subgroup analyses were conducted to explore its potential sources. Subgroup analysis of trials was performed according to the type of surgery (CABG alone, CABG combined with other cardiac surgery), administration time (preoperative only, from preoperative to postoperative, intraoperative only, from intraoperative to postoperative, postoperative only), route (oral, intravenous, both oral and intravenous), and dosage (low [< 2,000 mg], medium [2,000 to 3,000 mg], high [3,000 to 4,000 mg], very high [> 4,000 mg]).

The timing, route, and dosage of amiodarone administration varied substantially across trials, precluding systematic categorization by specific regimen. Therefore, the total cumulative dose of prophylactic administration was calculated for each study. For studies initiating prophylactic amiodarone postoperatively lasting for the entire ICU period or during hospitalization without reporting the administration duration, the mean ILOS or mean HLOS was used for calculation. If amiodarone was administered based on body weight, the average body weight reported in the study would be applied for dose calculation. The prescribing information for amiodarone tablets from the Food and Drug Administration (FDA) indicates an oral bioavailability of approximately 50% [18]. Assuming 100% bioavailability for the intravenous administration and referencing the method by Mitchell S Buckley et al. [19], all oral doses were converted to intravenous equivalents for comparative analysis through a bioavailability of 48%. Cumulative dose was measured and reported as an absolute value in milligrams (mg). Based on the defined ranges, amiodarone doses were categorized as low (< 2,000 mg), medium (2,000 to 3,000 mg), high (3,000 to 4,000 mg), or very high (> 4,000 mg).

Assessing the overlap of CI and performing a test for subgroup differences (the Chi^2^ test) is available in RevMan for POAF. A significance level of *P* < 0.05 for the interaction test will be considered suggestive of a potential subgroup effect. However, all subgroup findings will be interpreted as exploratory rather than confirmatory, given the observational nature of such analyses.

### Sensitivity analysis

The sensitivity was assessed to evaluate the individual studies’ influence on the overall effects, and the following pre-specified sensitivity analyses were performed: (1) sequential exclusion: repeating the meta-analysis by sequentially excluding each study to assess whether any single study disproportionately influences the overall effect estimate. (2) statistical model: comparing the results obtained from the random-effects model with those from a fixed-effect model. (3) methodological quality: restricting the analysis to studies’ overall bias judged to be low or with some concerns. (4) outcome definition: restricted to studies reporting POAF as the sole outcome (excluding AFL or SVT) and restricted to studies with a complete POAF definition (reported POAF as the sole outcome [excluding AFL or SVT] and provided a complete definition specifying episode duration, diagnostic criteria, and monitoring duration). (5) monitoring methods: included studies that employed specified-duration continuous electrocardiograph (ECG)/Holter monitoring. A result will be considered robust if the effect estimates from these sensitivity analyses do not differ materially in direction, magnitude, or statistical significance from the primary analysis.

### Certainty of the evidence assessment

The overall certainty of the evidence for each pre-specified critical and important outcome was assessed using the Grading of Recommendations Assessment, Development and Evaluation (GRADE) approach for both the main analysis (including all studies) and the sensitivity analysis restricted to studies at low risk or some concerns by two researchers (Li Z and An L) independently using GRADEpro GDT software [20]. Under the GRADE framework, evidence from RCTs initially is assigned a high certainty rating. This rating may subsequently be downgraded for limitations within five key domains: (1) risk of bias (study limitations); (2) inconsistency (unexplained heterogeneity); (3) indirectness of evidence; (4) imprecision of the effect estimates; and (5) publication bias. The certainty of the evidence is graded into one of four levels: (1) high: the true effect lies close to the estimated one; (2) moderate: the true effect is likely to be close to the estimated one or possibly to be substantially different; (3) low: the true effect may be substantially different from the estimated one; (4) very low: the true effect is likely to be substantially different from the estimated one. Any disagreements will be resolved through discussion or by consulting a third author (Chen C).

### Meta-regression

Meta-regression analyses were conducted to explore sources of heterogeneity and the dose–response relationship, using mixed-effects models with restricted maximum likelihood (REML) estimation in R software (package metafor). And all data visualization plots were generated in R software (package ggplot2).

For the univariable meta-regression, we regressed the log odds ratio (log OR) on the cumulative dose (mg) alone. For the multivariate meta-regression, we extended the model to include timing (preoperative only, from preoperative to postoperative, intraoperative only, from intraoperative to postoperative, postoperative only) and route (oral, intravenous, both oral and intravenous) as categorical moderators. Reference levels were set to postoperative for timing and intravenous for route. The proportion of heterogeneity explained was calculated as *R*^2^. Based on the multivariate results, we performed subgroup-specific dose–response regressions for some strategy combinations: from preoperative to postoperative + oral, postoperative only + both oral and intravenous, and postoperative only + intravenous (requiring ≥ 2 studies per subgroup). For all models, we reported the regression coefficient (β), 95% CI, P-value, heterogeneity measures (Tau^2^, I^2^), and model test statistics (QM, QE). Significance was set at *P* < 0.05.

## Results

### Results of search strategy and selection

**Fig. 1:**
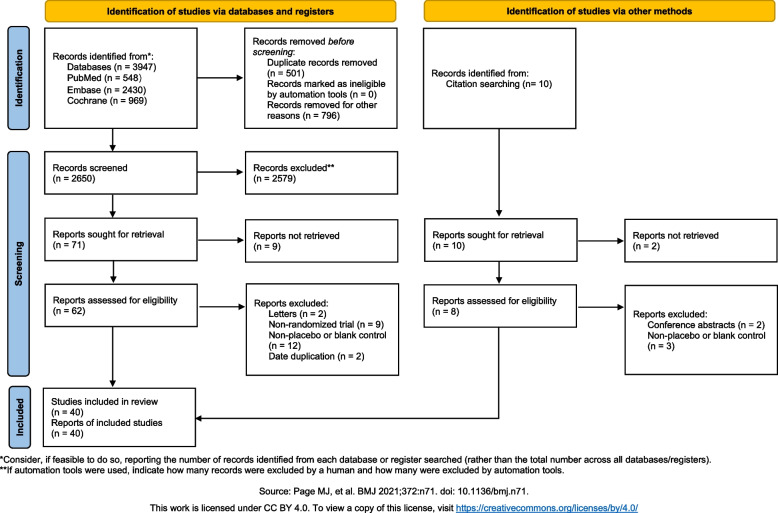
PRISMA flow diagram

The PRISMA flowchart is shown in Fig. 1, containing the study selection process of screening and evaluating. After searching databases (PubMed, Embase, and the Cochrane Library), we revealed a total of 3,446 unique studies. A further 10 studies were collected by filtering references from previous studies. 2,579 studies were excluded based on titles and abstracts, and 81 studies met the inclusion criteria. Except for 11 studies that were not available in full text or whose main text was written in a non-English language, the remaining 70 studies were obtained in full text. After reviewing the full text, we identified 40 studies and included them in the final analysis [21–60].

### Characteristics of the included studies

The studies’ characteristics were summarized in Tables 1, 2, and 3. There are 40 studies of 6,166 patients comparing amiodarone with placebo or no treatment [21–60]. Among those studies, 35 studies (87.5%) were published between 1991 and 2011 [21–55], and only two studies were published in the past 5 years [59, 60].

**Table 1:** Baseline characteristics of the included studies

Author (Year)	Journal	Study design	Surgery	Other criteria^a^	Sample size	Intervention size	Control size	Groups
S H Hohnloser et al. (1991) [21]	Am Heart J	RCT	CABG	Not reported	77	39	38	Amiodarone vs. placebo
J Butler et al. (1993) [22]	Br Heart J	RCT	CABG	Not reported	120	60	60	Amiodarone vs. placebo
Emile G Daoud et al. (1997) [23]	N Engl J Med	RCT	CABG, CVS	Not reported	124	64	60	Amiodarone vs. placebo
Thomas Guarnieri et al. (1999) [24]	J Am Coll Cardiol	RCT	CABG, CVS, ASD	Not reported	300	158	142	Amiodarone vs. placebo
Joseph D Redle et al. (1999) [25]	Am Heart J	RCT	CABG	Not reported	143	73	70	Amiodarone vs. placebo
M M Treggiari-Venzi et al. (2000) [28]	Br J Anaesth	RCT	CABG	Not reported	147	49	51	Amiodarone vs. placebo vs. magnesium
Shih-Huang Lee et al. (2000) [27]	Ann Thorac Surg	RCT	CABG	Not reported	150	74	76	Amiodarone vs. placebo
Hilmar Dörge et al. (2000) 1 [26]	Ann Thorac Surg	RCT	CABG	Not reported	150	50	25	Amiodarone vs. placebo
Hilmar Dörge et al. (2000) 2 [26]	Ann Thorac Surg	RCT	CABG	Not reported	150	50	25	Amiodarone vs. placebo
Dejan Maraš et al. (2001) [31]	Am Heart J	RCT	CABG	Not reported	315	159	156	Amiodarone vs. placebo
Basel S Harahsheh et al. (2001) [30]	Saudi Med J	RCT	CABG	Not reported	180	88	92	Amiodarone vs. placebo
Satyendra Giri et al. (2001) [29]	Lancet	RCT	CABG, CVS	Not reported	220	120	100	Amiodarone vs. placebo
Alexandre Yazigi et al. (2002) [34]	J Cardiothorac Vasc Anesth	RCT	CABG	Not reported	200	100	100	Amiodarone vs. placebo
C Michael White et al. (2002) 1 [33]	Ann Thorac Surg	RCT	CABG, CVS	≥ 60 years old	200	56	50	Amiodarone vs. placebo
C Michael White et al. (2002) 2 [33]	Ann Thorac Surg	RCT	CABG, CVS	≥ 60 years old	200	64	50	Amiodarone vs. placebo
Hilmi Tokmakoglu et al. (2002) [32]	Eur J Cardiothorac Surg	RCT	CABG	Not reported	241	72	92	Amiodarone vs. no-treatment vs. metoprolol + digoxin
Eugene Crystal et al. (2003) 1 [35]	J Thorac Cardiovasc Surg	RCT	CABG, CVS, Aneurysm resection	24-h Holter monitor, 10 VPBs/h or at least 1 VT	82	24	23	Amiodarone vs. placebo
Eugene Crystal et al. (2003) 2 [35]	J Thorac Cardiovasc Surg	RCT	CABG, CVS, Aneurysm resection	24-h Holter monitor, 10 VPBs/h or at least 1 VT	82	12	23	Amiodarone vs. placebo
C Michael White et al. (2003) [36]	Circulation	2 × 2 factorial design	CABG, CVS	≥ 50 years old	160	77	83	Amiodarone vs. placebo
Tahir Yagdi et al. (2003) [37]	J Thorac Cardiovasc Surg	RCT	CABG	Not reported	157	77	80	Amiodarone vs. no-treatment
Eigil Nygård et al. (2004) [39]	J Cardiothorac Vasc Anesth	2 × 2 factorial design	CABG	Not reported	163	36	48	Amiodarone vs. no-treatment vs. TEA vs. TEA + amiodarone
Erkan Kuralay et al. (2004) [38]	Eur J Cardiothorac Surg	RCT	CABG	Not reported	300	100	100	Amiodarone vs. no-treatment
L Brent Mitchell et al. (2005) [40]	JAMA	RCT	CABG, CVS	≥ 18 years old	601	299	302	Amiodarone vs. placebo
Rafael Vieira Alcalde et al. (2006) [41]	Arq Bras Cardiol	RCT	MRS	Not reported	93	46	47	Amiodarone vs. placebo
Kerim Cagli et al. (2006) [43]	J Card Surg	RCT	CABG	≥ 3 high-risk criteria^b^	136	44	48	Amiodarone vs. placebo vs. amiodarone + magnesium
Marco Budeus et al. (2006) [42]	Eur Heart J	RCT	CABG	Not reported	110	55	55	Amiodarone vs. placebo
Tamer Turk et al. (2007) [45]	Heart Surg Forum	RCT	CABG	Not reported	144	76	68	Amiodarone vs. placebo
Lars R Zebis et al. (2007) [46]	Ann Thorac Surg	RCT	CABG	Not reported	223	113	110	Amiodarone vs. placebo
Renato Jorge Alves et al. (2007) [44]	Arq Bras Cardiol	RCT	CABG, CVS	≥ 3 high-risk criteria^c^	68	15	20	Amiodarone vs. no-treatment
Lars R Zebis et al. (2008) [48]	Ann Thorac Surg	RCT	CABG	Not reported	250	125	125	Amiodarone vs. placebo
Craig I Coleman et al. (2008) [47]	Expert Opin Pharmacother	2 × 2 factorial design study	CABG	≥ 50 years old	180	26	64	Amiodarone vs. no-treatment
Song Gu et al. (2009) [50]	Chin Med J (Engl)	RCT	CABG	≥ 70 years old	210	100	110	Amiodarone vs. placebo
F Akbarzadeh et al. (2009) [49]	Pak J Biol Sci	RCT	CABG	Not reported	211	70	70	Amiodarone vs. no-treatment vs. atrial pacing
Farideh Roshanali et al. (2009) [51]	Interact Cardiovasc Thorac Surg	RCT	CABG	AEMi > 120 ms	100	50	50	Amiodarone vs. placebo
Osman Tiryakioglu et al. (2009) [52]	J Cardiothorac Surg	RCT	CABG	Not reported	192	64	64	Amiodarone vs. placebo vs. magnesium
Yasser Mohamed Amr et al. (2010) [53]	Anesth Essays Res	RCT	CVS	Not reported	97	46	48	Amiodarone vs. placebo
Yanick Beaulieu et al. (2010) [54]	Anesthesiology	RCT	CABG, CVS	LVEF ≥ 20%	120	60	60	Amiodarone vs. placebo
Kar Sandeep Kumar et al. (2011) [55]	Ann Card Anaesth	RCT	CVS	Not reported	56	28	28	Amiodarone vs. placebo
Xiao Dong Feng et al. (2014) [56]	J Thorac Cardiovasc Surg	RCT	CABG	Not reported	100	50	50	Amiodarone vs. no-treatment
Moshiri Esmail et al. (2015) [57]	J Cardiovasc Dis Res	RCT	CABG	ASA physical status class II and III, 2 or 3 grafts	124	62	62	Amiodarone vs. placebo
William Wang et al. (2016) [58]	J Thorac Cardiovasc Surg	RCT	CABG	Not reported	150	50	50	Amiodarone vs. placebo vs. triamcinolone
O L Bockeria et al. (2020) [59]	J Cardiovasc Transl Res	RCT	CABG	Not reported	60	30	30	Amiodarone vs. no-treatment
Mohamed Ali Ahmed et al. (2022) [60]	The Cardiothoracic Surgeon	RCT	CABG	Not reported	150	75	75	Amiodarone vs. placebo

*Abbreviations*: *AEMi* Atrial electromechanical interval, *ASA* American Society of Anesthesiologists, *ASD* Atrial septal defect, *CABG* Coronary artery bypass grafting, *CVS* Cardiac valve surgery, *LVEF* Left ventricular ejection fraction, *MRS* Myocardial revascularization surgery, *RCT* Randomized controlled trial, *TEA* Thoracic epidural anesthesia, *VPB* Ventricular premature beats, *VT* Ventricular tachycardia

^a^Other inclusion criteria, in addition to the requirements for sinus rhythm, hemodynamic stability, and absence of atrial fibrillation history

^b^The high-risk criteria included age ≥ 65 years, male sex, history of hypertension or diabetes mellitus, need for early postoperative inotropic medication or intra-aortic balloon counterpulsation, left ventricular end-diastolic pressure greater than 18 mmHg, and decreased left ventricular ejection fraction < 50%

^c^The high-risk criteria included age > 65 years, valve disease, left atrium overload, ventricular dysfunction, cardiac reoperation, electrolyte disorder, previous atrial arrhythmia, hypoxemia, chronic obstructive pulmonary disease, discontinuation of betablocker 24 h before surgery, and previous use of digitalis

**Table 2:** Summary of amiodarone intervention protocols

Author (Year)	Route	Stage	Timing	Dosing regimen	Planned dose^a^	Reported dose^b^
S H Hohnloser et al. (1991) [21]	IV	Post	Post immediately to 4d	Postoperative, 300 mg, IV, within 2 h, then 1.2 g/24 h, IV, for 2 d, then 900 mg/24 h, IV, for 2d	4500 mg	Not reported
J Butler et al. (1993) [22]	IV and PO	Intra to post	Intra to post 5d	Intraoperative, 15 mg/kg (max 1500 mg), IV, for 24 h. Postoperative, 200 mg, PO, TID, for 5d	2940 mg^c^	Not reported
Emile G Daoud et al. (1997) [23]	PO	Pre to post	Pre 13 ± 7 d to post discharge	Preoperative, start at 13 ± 7 d, 200 mg, PO, TID, for 7d. Postoperative, 200 mg, PO, QD, to discharge. For patients being treated with digitalis or warfarin, the doses were halved	2640 mg	2304 mg
Thomas Guarnieri et al. (1999) [24]	IV	Post	Post in ICU within 3 h to 48 h	Postoperative, start in ICU within 3 h, 1 g/d, IV, for 48 h	2000 mg	Not reported
Joseph D Redle et al. (1999) [25]	PO	Pre to post	Pre 1-4d to post 7d	Preoperative, if start at 4 d prior surgery, 200 mg, PO, BID, for 5 d (including surgery day), if start at 3 d prior surgery, 200 mg, BID, PO, for 2 d, then 200 mg, PO, TID, for 2 d (including surgery day), if start at 2 d prior surgery, 200 mg, PO, TID, for 2 d, then 200 mg, PO, QID, on surgery day, if start at 1 d prior surgery, 200 mg, PO, PID, for 2 d (including surgery day). Postoperative, 200 mg, PO, BID, for 7d	2304 mg	Not reported
M M Treggiari-Venzi et al. (2000) [28]	IV	Post	Post in ICU within 1 h to 3d	Postoperative, 900 mg/d, IV, start in ICU within 1 h, for 3d	2700 mg	Not reported
Shih-Huang Lee et al. (2000) [27]	IV	Pre to post	Pre 3 d to post 5d	Preoperative, loading 150 mg, IV, then 0.4 mg/kg/h, IV, for 3d. Postoperative, 0.4 mg/kg/h, IV, for 5d	NA^d^	Not reported
Hilmar Dörge et al. (2000) 1 [26]	IV	Intra to post	Intra to post 3d	Intraoperative, 300 mg, IV, then 20 mg/kg/d, IV, for 3d. (300 mg loading dose group)	NA^d^	Not reported
Hilmar Dörge et al. (2000) 2 [26]	IV	Intra to post	Intra to post 3d	Intraoperative, 150 mg, IV, then 10 mg/kg/d, IV, for 3d. (150 mg loading dose group)	NA^d^	Not reported
Dejan Maraš et al. (2001) [31]	PO	Pre to post	Pre 1 d to post 6d	Preoperative, 1200 mg, PO, QD, for 1d. Postoperative, 200 mg, PO, QD, for 7 d, including the surgery day	1248 mg	Not reported
Basel S Harahsheh et al. (2001) [30]	IV and PO	Post	Post in ICU to 4w	Postoperative, dosage unknown, IV continue for 24 h, then 200 mg, PO, TID, for 1w, then 200 mg PO, BID, for 1w, then QD, PO, for 4w	NA^e^	Not reported
Satyendra Giri et al. (2001) [29]	PO	Pre to post	Pre 1-5d to post 4d Pre 1 d to post 4d	Preoperative, 200 mg, PO, TID, start > 5 d before surgery, for 5d. Surgery day, 400 mg, PO, BID. Postoperative, 400 mg, PO, BID, for 4d. (slow loading group) Preoperative, 400 mg, PO, QID, for 1d. Surgery day, 600 mg, PO, BID. Postoperative, 400 mg, PO, BID, for 4d. (fast loading group)	3360 mg 2880 mg	2563 mg^f^
Alexandre Yazigi et al. (2002) [34]	PO	Post	Post in ICU within 4 h to discharge	Postoperative, in ICU within 4 h, 15 mg/kg/d, PO, for 1 d, then 7 mg/kg/d, PO, until discharge	2238 mg	Not reported
C Michael White et al. (2002) 1 [33]	PO	Pre to post	Pre > = 5 d or < 5 d but > 1 d prior to surgery to post 4d	Preoperative, start ≥ 5 d prior to surgery, 200 mg, PO, TID, for 5d. Surgery day, 400 mg, PO, BID. Postoperative, 400 mg, PO, BID, for 4d. (slow loading group)	3360 mg	Not reported
C Michael White et al. (2002) 2 [33]	PO	Pre to post	Pre > = 5 d or < 5 d but > 1 d prior to surgery to post 4d	Preoperative, start < 5 d but > 1 d prior to surgery, 400 mg, PO, QID, for 1d. Surgery day, 600 mg, PO, BID. Postoperative, 400 mg, PO, BID, for 4d. (fast loading group)	2880 mg	Not reported
Hilmi Tokmakoglu et al. (2002) [32]	IV and PO	Post	Post within 1 h to discharge	Postoperative, 300 mg, IV, within 1 h, then 900 mg, IV, for 24 h; then 450 mg, IV, for 24 h, then 200 mg, PO, TID, until discharge	NA^g^	Not reported
Eugene Crystal et al. (2003) 1 [35]	PO	Pre to post	Pre to post	Preoperative, 10 mg/kg/d, PO, for 2w, then 400 mg/d, PO. Patients ≤ 60 kg or ≥ 75 years, reduced to 300 mg/d. If arrhythmia suppression was observed at both 4 m and 8 m, reduced by 100 mg/d to a potential minimum dose of 200 mg/d, patients ≤ 60 kg or ≥ 75 years, 200 mg/d 5 d/w. Postoperative, for 7 d, 353 ± 84 mg. (active group)	NA^d^	Not reported
Eugene Crystal et al. (2003) 2 [35]	PO	Post	Pre	Preoperative, 10 mg/kg/d, PO, for 2w, then 400 mg/d, PO. Patients ≤ 60 kg or ≥ 75 years, reduced to 300 mg/d. If arrhythmia suppression was observed at both 4 m and 8 m, reduced by 100 mg/d to a potential minimum dose of 200 mg/d, patients ≤ 60 kg or ≥ 75 years, 200 mg/d 5 d/w. Preoperative, discontinue 15 d (11d-41d). (discontinue group)	NA^d^	Not reported
C Michael White et al. (2003) [36]	IV and PO	Post	Post within 6 h to 5d	Postoperative, 1050 mg, IV, within 6 h, then 400 mg, PO, TID, for 4d. Equivalent of 6900 mg oral drug	3354 mg	2701 mg
Tahir Yagdi et al. (2003) [37]	IV and PO	Post	Post within 2 h to 30d	Postoperative, start within 2 h, 10 mg/kg/d, IV, for 48 h, then 200 mg, PO, TID, for 5 d, then 200 mg, PO, BID, for 5 d, then 200 mg, PO, QD, for 20d	NA^d^	Not reported
Eigil Nygård et al. (2004) [39]	PO and IV	Pre to post	Pre 1 d to post 3d	Preoperative, 1800 mg, PO, QD. Intraoperative, 900 mg/d, IV, after anesthesia induction to postoperative 3d	3564 mg	Not reported
Erkan Kuralay et al. (2004) [38]	IV and PO	Post	Post in ICU to 4 m	Postoperative, for intubated, 1200 mg/d, IV, for 1 d, then 400 mg/d, IV, for 10 d, then 400 mg/d, PO, for 10 d, then 200 mg/d, for 4 m; for not intubated, 1200 mg/d, IV, for 1 d, then 600 mg/d, PO, for 10 d, then 400 mg/d PO, for 10 d, then 200 mg/d, PO, for 4 m	18640 mg	Not reported
L Brent Mitchell et al. (2005) [40]	PO	Pre to post	Pre 6 d to post 6 d, a total of 13d	Preoperative, 10 mg/kg, PO, BID, for 6d. Postoperative, 10 mg/kg, PO, BID, for 6d. A total of 13d	NA^d^	Not reported
Rafael Vieira Alcalde et al. (2006) [41]	PO	Pre	Pre 30–56 h to surgery day	Preoperative, 300 mg, PO, TID, to surgery day	1296 mg	1248 mg
Kerim Cagli et al. (2006) [43]	IV	Intra	Intra	Intraoperative, 5 mg/kg, IV, for 30 min	NA^d^	Not reported
Marco Budeus et al. (2006) [42]	PO and IV	Pre to post	Pre 1 d to post 7d	Preoperative, 200 mg, PO, TID. Postoperative, 300 mg, IV, for 1 h for loading dose, then 20 mg/kg, IV, for 24 h, then 200 mg, PO, TID, from day2 to day7	2371 mg h	Not reported
Tamer Turk et al. (2007) [45]	IV and PO	Post	Post 1 d to 30d	Postoperative, 5 mg/kg, IV, for 24 h, then 200 mg, PO, TID, 600 mg/d, for 30d	NA^d^	Not reported
Lars R Zebis et al. (2007) [46]	IV and PO	Post	Post 1 d to 5d	Postoperative, 300 mg, IV, 8 AM, for 20 min, then 600 mg, PO for the first dose, then 600 mg, PO, BID, for 5d. Total dose of 6.3 g	3468 mg	3024 mg
Renato Jorge Alves et al. (2007) [44]	IV and PO	Post	Post 1 d to 7 d or discharge	Postoperative, 600–900 mg, IV, within 24 h, then 400 mg/d, PO, for 7 d or discharge	2094 mg	Not reported
Lars R Zebis et al. (2008) [48]	IV and PO	Post	Post 1 d to 5d	Postoperative, 8 AM at ICU, 300 mg, IV, for 20 min, then 600 mg, PO, BID, 8 AM and 5 PM, for 5d	3468 mg	3024 mg
Craig I Coleman et al. (2008) [47]	IV and PO	Not reported	Not reported	Not reported	NA^i^	Not reported
Song Gu et al. (2009) [50]	PO	Pre to post	Pre 7 d to post 10 d	Preoperative, 10 mg/kg/d, PO, for 7d. Postoperative, 0.2 g (1.5–3.0 mg/kg), for 10d	3648 mg	Not reported
F Akbarzadeh et al. (2009) [49]	IV	Post	Post on arrival to the ICU to discharge	Postoperative, on arrival to ICU, 150 mg, IV, over 30 min, then 1 mg/h for 6 h, then 0.5 mg/h, until discharge	NA^g^	Not reported
Farideh Roshanali et al. (2009) [51]	PO and IV	Pre to post	Pre 1 d to post 5d	Preoperative, 800 mg, PO, for 1d. Intraoperative, 500 mg, IV, for 1 h. Postoperative, 20 mg/kg/d, IV, for 1 d, then 800 mg/d, PO, from day2 to day5	NA^c^	Not reported
Osman Tiryakioglu et al. (2009) [52]	IV and PO	Post	Post 1 d to 3d	Postoperative, 1200 mg, IV, the first day, then 600 mg/d, PO, the day2 and day3	1776 mg	Not reported
Yasser Mohamed Amr et al. (2010) [53]	IV	Intra	Intra	Intraoperative, prior to making skin incision, 3 mg/kg, IV, diluted in 100 mL of normal saline, over 30 min	225 mg	Not reported
Yanick Beaulieu et al. (2010) [54]	IV	Intra to post	Intra continue for 48 h	Intraoperative, after anesthesia induction, loading dose of 300 mg, IV, over 10 min, then 15 mg/kg/d, IV, for 48 h, with max daily dose of 1500 mg/d	3300 mg^c^	2744 mg
Kar Sandeep Kumar et al. (2011) [55]	IV	Intra	Intra	Intraoperative, 20 min before CPB, 3 mg/kg, IV	153 mg	Not reported
Xiao Dong Feng et al. (2014) [56]	Hydrogel material	Intra	Intra	Intraoperatively, before the sternum was closed, spraying hydrogels diffusely onto the exposed epicardial surfaces over the right atrial lateral wall, left atrial appendage, and transverse sinus area, amiodarone hydrochloride powder 1 mg/kg	NA^d^	Not reported
Moshiri Esmail et al. (2015) [57]	IV	Intra to post	Intra to post 24 h	Intraoperatively, after performing an arterial line, 300 mg, IV, within 20–30 min, postoperatively, 1 mg/kg IV, for 6 h, then 0.5 mg/kg, IV, for 18 h	NA^d^	Not reported
William Wang et al. (2016) [58]	Hydrogel material	Intra	Intra	Intraoperatively, before the sternum was closed, spraying hydrogels diffusely onto the exposed epicardial surfaces over the right atrial lateral wall, left atrial appendage, and transverse sinus area, amiodarone hydrochloride powder 1 mg/kg	NA^d^	Not reported
O L Bockeria et al. (2020) [59]	Hydrogel material	Intra	Intra	Intraoperatively, on termination of CPB, apply biatrial epicardial amiodarone-releasing hydrogel, spraying on the surface of both atria in the amount of 5–8 ml, at a concentration calculated as 1 mg/kg	NA^d^	Not reported
Mohamed Ali Ahmed et al. (2022) [60]	PO	Pre to post	Pre 6 d to post 6d	Preoperative, 5 mg/kg divided into 2 doses/d, PO, for 6 d; postoperative, same doses, PO, for 6d	NA^d^	Not reported

Units of measurement: mg, milligram; kg, kilogram; min, minute; h, hour; d, day; w, week; m, month

*Abbreviations*: *BID* Bis in die (twice daily), *CPB* Cardiopulmonary bypass, *ICU* Intensive care unit, *Intra* Intraoperative, *IV* Intravenous, *NA* Not available, *PO* Per os (orally), *Post* postoperative, *Pre* Preoperative, *QD* Quaque die (once daily), *QID* Quater in die (four times daily), *TID* Ter in die (three times daily)

^a^Calculations were based on the dosing regimens described in the Methods sections and relevant data in the Results sections of the included studies. Any missing parameters required for these calculations were documented as NA

^b^Calculations were based on the dosage reported in the Results sections of the included articles. In cases where both a planned dose and a reported dose were available, the reported dose was used for the classification of dosage strength

^c^In the absence of reported body weight, the dosage was computed based on the maximum value defined in the dosing regimen

^d^Due to the absence of body weight data, the total cumulative dose could not be calculated

^e^Due to the absence of intravenous dosing regimen, the total cumulative dose could not be calculated

^f^For studies that stratified patients into rapid and slow loading groups, the outcomes were not analyzed separately, and a single drug dose was reported

^g^Due to the lack of reported hospital length of stay, the total cumulative dose could not be calculated

^h^For this study with unavailable body weight, the dose was estimated using the total amiodarone cost and unit price reported in the study

^i^Due to the absence of the dosing regimen, the total cumulative dose could not be calculated

**Table 3:** Definitions and monitoring methods for postoperative atrial fibrillation

Author (Year)	Primary outcome	POAF duration	Monitoring duration	POAF definition	POAF monitor
S H Hohnloser et al. (1991) [21]	VA	Not reported	48 h	SVT (requiring therapeutic intervention)	Holter monitoring (for 48 h); ECG (at 2,6,12,24,48 h, after 48 h, for symptomatic arrhythmias)
J Butler et al. (1993) [22]	SVT	5 min	6d	AF or AFL (> 1 min); SVT (> 5 min, > 100 bpm; requiring treatment)	Continuous Holter monitoring (for 6 d); 12-lead ECG (at 1,3,5d and if clinically detected)
Emile G Daoud et al. (1997) [23]	POAF	5 min	In hospital	AF (> 5 min)	3-lead telemetric monitoring (7.2 ± 3.6d)
Thomas Guarnieri et al. (1999) [24]	POAF and LOS	Not reported	30d	AF (requiring any treatment)	Continuous ambulatory monitoring
Joseph D Redle et al. (1999) [25]	POAF	30 min	4d	AF (≥ 30 min; requiring treatment for symptoms/hemodynamic compromise)	Continuous ECG monitoring (for ≥ 96 h)
M M Treggiari-Venzi et al. (2000) [28]	POAF	30 s	3d	AF (irregular atrial rhythm); SVT (> 3 complexes, > 100 bpm, > 30 s)	Holter ECG (for 72 h); 12-lead ECG (every 12 h)
Shih-Huang Lee et al. (2000) [27]	POAF	10 min	In hospital	AF (> 10 min; atrial activity not discernible or completely unorganized, with irregular ventricular rate)	Continuous monitoring (in ICU; in ward); 12-lead ECG (confirmation)
Hilmar Dörge et al. (2000) 1 [26]	POAF	5 min	10d	AF (> 5 min)	Continuous ECG monitoring (for 10 d); 12-lead ECG (day 1, pre-discharge)
Hilmar Dörge et al. (2000) 2 [26]	POAF	5 min	10d	AF (> 5 min)	Continuous ECG monitoring (for 10 d); 12-lead ECG (day 1, pre-discharge)
Dejan Maraš et al. (2001) [31]	POAF	60 min	7d	AF (> 60 min; with hemodynamic compromise)	Continuous monitoring (in ICU, ≥ 24 h); ECG (after 24 h, 10 AM and 5 PM twice daily; if symptomatic [irregular pulse, heart rate > = 100 bpm, hypotensive, angina, dyspnea, sweating]); radial pulse check (6 times daily)
Basel S Harahsheh et al. (2001) [30]	POAF	Not reported	6w	Not reported	12-lead ECG (at 6w)
Satyendra Giri et al. (2001) [29]	POAF	5 min	30d	AF (> 5 min)	Continuous telemetry; 12-lead ECG (daily; if symptomatic)
Alexandre Yazigi et al. (2002) [34]	POAF	5 min	In hospital	AF (> 5 min; clinically suspected and ECG confirmed)	5-lead alarm-triggered ECG scope (in ICU); 12-lead ECG (twice daily); nurse evaluation (q4h)
C Michael White et al. (2002) 1 [33]	POAF	5 min	30d	AF (> 5 min)	Continuous ECG telemetry (in ICU; in ward); 12-lead ECG (daily; if symptomatic)
C Michael White et al. (2002) 2 [33]	POAF	5 min	30d	AF (> 5 min)	Continuous ECG telemetry (in ICU; in ward); 12-lead ECG (daily; if symptomatic)
Hilmi Tokmakoglu et al. (2002) [32]	POAF	Not reported	In hospital	Not reported	Continuous monitoring (in ICU, for 48 h); Continuous ECG monitoring (in ward, q30min, until discharge)
Eugene Crystal et al. (2003) 1 [35]	Postoperative complications	30 min	7d	AF (≥ 30 min)	ECG monitoring (for 7 d)
Eugene Crystal et al. (2003) 2 [35]	Postoperative complications	30 min	7d	AF (≥ 30 min)	ECG monitoring (for 7 d)
C Michael White et al. (2003) [36]	POAF	5 min	30d	AF (> 5 min; symptomatic if hemodynamic compromise [hypotension, heart failure] requiring treatment or subjective discomfort [palpitations, chest pain, shortness of breath, syncope])	Telemetry (with 24 h storage); 12-lead ECG (daily)
Tahir Yagdi et al. (2003) [37]	POAF	5 min	30d	AF (> 5 min, discernible or completely with irregular ventricular rate; requiring therapy for hemodynamic compromise)	Continuous ECG telemetry (in ICU; in ward); 12-lead ECG (daily; if detected)
Eigil Nygård et al. (2004) [39]	POAF	5 min	5d	AF (> 5 min; irregular narrow complex rhythm with absence of P waves)	3-lead 24-h Holter recordings (for 5 d)
Erkan Kuralay et al. (2004) [38]	SVT	Not reported	In hospital	Not reported	Continuous monitoring (in ICU); radial pulse check (6 times daily)
L Brent Mitchell et al. (2005) [40]	Atrial tachyarrhythmia	5 min	6d	Atrial tachyarrhythmia (≥ 5 min; requiring treatment)	Continuous telemetry ECG monitoring (for 6 d)
Rafael Vieira Alcalde et al. (2006) [41]	POAF	10 min	In hospital	AF/AFL (≥ 10 min; if shorter with hemodynamic instability); AF (the absence of P waves before each QRS complex and an irregular ventricular frequency); AFL (the absence of P waves, presence of biphasic F waves, with either fixed or variable atrioventricular block)	Continuous ECG monitoring (in ICU); 12-lead ECG (in ward, daily; if symptomatic palpitations and/or suspected arrhythmia)
Kerim Cagli et al. (2006) [43]	POAF	30 min	In hospital	AF (> 30 min; requiring treatment for symptoms/hemodynamic compromise)	Continuous ECG monitoring (in ICU, for ≥ 48 h); 12-lead ECG (in ward, every 8 h; if symptomatic [irregular pulse, heart rate > = 100 bpm, hypotensive, angina, dyspnea, sweating])
Marco Budeus et al. (2006) [42]	POAF	10 min	7d	AF (> 10 min)	Continuous Holter monitoring (for 7 d)
Tamer Turk et al. (2007) [45]	POAF	10 min	In hospital	AF (> 10 min)	Continuous ECG (for 72 h); 12-lead ECG (at 2 h; daily until discharge); Nurse evaluation (q4h)
Lars R Zebis et al. (2007) [46]	POAF	30 min	30d	Asymptomatic AF (> 30 min, irregular pulse during ward rounds); symptomatic AF (with discomfort or hemodynamic compromise, palpitations, angina pectoris, shortness of breath, or fainting, or the need for acute intervention owing to hypotension or heart failure, ECG confirmed); AF (fast, irregular, eddy current activation of the atrium with neutralization of its contractions; the ECG shows totally irregular ventricle rhythm with a constant irregular fluctuation of the length between the QRS-complexes, the QRS-complexes often appear with a frequency of about 150 to 200 beats/min, displaying a line flickering with narrow QRS-complexes)	Continuous monitoring (in ICU); 12-lead ECG (daily for 5 d; at 30 d follow-up; if clinical signs [irregular pulse, fainting, confusion, shortness of breath] or subjective manifestations [palpitations, dizziness, fatigue, light-headedness])
Renato Jorge Alves et al. (2007) [44]	POAF	Not reported	In hospital	Not reported	ECG and/or cardiac monitoring
Lars R Zebis et al. (2008) [48]	Cost	Not reported	5d	AF (fast irregular, eddy current activation of the atrium with neutralization of its contractions)	Continuous telemetry monitoring (in ICU); 12-lead ECG (in ward; daily; if symptomatic)
Craig I Coleman et al. (2008) [47]	POAF	5 min	30d	AF (> 5 min)	Not reported
Song Gu et al. (2009) [50]	POAF	10 min	14d	AF (> 10 min)	Continuous ECG (for 7 d); 12-lead ECG (daily until 14 d)
F Akbarzadeh et al. (2009) [49]	POAF	60 min	In hospital	AF (> 60 min; with hemodynamic compromise)	Continuous telemetry monitoring (in ICU); 12-lead ECG (in ICU, daily, at discharge)
Farideh Roshanali et al. (2009) [51]	POAF and ICU stay	5 min	In hospital	AF (> 5 min, requiring treatment); Brief isolated non-sustained episodes of AF were ignored	Continuous ECG telemetry (until discharge); 12-lead ECG (daily)
Osman Tiryakioglu et al. (2009) [52]	POAF	Not reported	3d	Not reported	12-lead ECG (at 0,6,12 h and 1,2,3 d)
Yasser Mohamed Amr et al. (2010) [53]	POAF	5 min	5d	AF (> 5 min; regardless of the effect on the hemodynamic status or the need for medication)	Continuous ECG monitoring (for 5 d); 12-lead ECG (daily for 5 d; at discharge)
Yanick Beaulieu et al. (2010) [54]	POAF	30 min	In hospital	AF (> 30 min; requiring urgent treatment because of associated hemodynamic compromise (heart failure, hypotension, and ischemia) or symptomatic discomfort (shortness of breath, palpitations, and chest pain))	3-lead continuous Holter monitoring (for 4 d); Telemetry monitoring (until discharge); 12-lead ECG (daily)
Kar Sandeep Kumar et al. (2011) [55]	POAF	Not reported	Not reported	Not reported	Not reported
Xiao Dong Feng et al. (2014) [56]	POAF	Not reported	14d	Not reported	Continuous telemetry monitoring (daily until 14 d)
Moshiri Esmail et al. (2015) [57]	POAF	Not reported	2d	Not reported	Not reported
William Wang et al. (2016) [58]	POAF	Not reported	14d	Not reported	Continuous telemetry monitoring (daily until 14 d)
O L Bockeria et al. (2020) [59]	POAF	5 min	5d	AF (> 5 min)	3-lead Holter ECG monitoring (for 72 h); 12-lead ECG (twice daily after 72 h); 24 h Holter (on day 5)
Mohamed Ali Ahmed et al. (2022) [60]	POAF	5 min	6d	AF (≥ 5 min, persistent P waves did not precede every QRS complex, and the ventricular rate was abnormal)	Continuous ECG monitoring (for 6 d)

Units and frequencies: bpm, beats per minute; min, minute; h, hour; d, day; qXh, every X hours (e.g., q4h, every 4 h)

*Abbreviations*: *AF* Atrial fibrillation, *AFL* Atrial flutter, *ECG* Electrocardiogram, *ICU* Intensive care unit, *LOS* Length of stay, *POAF* Postoperative atrial fibrillation, *SVT* Supraventricular tachycardia, *VA* Ventricular arrhythmia

The weighted mean age of trial participants in the intervention and control groups was 63.6 ± 7.4 years and 64.1 ± 7.9 years, respectively, with males accounting for 78.0% and 77.3% of each group. The respective weighted LVEF was 56.4 ± 3.8% of the intervention group and 56.6 ± 5.4% of the control group.

Regarding the types of surgery, among the 40 studies, 29 (72.5%) involved isolated CABG [21, 22, 25–28, 30–32, 34, 37–39, 41–43, 45–52, 56–60], while 9 (22.5%) involved CABG and/or other types of cardiac surgery (primarily referring to valve surgery) [23, 24, 29, 33, 35, 36, 40, 44, 54]. Regarding the controls, a placebo was administered in 31 (77.5%) studies, while no treatment was used in the remaining 9 (22.5%) studies.

The studies by Hilmar Dörge et al. (2000) [26], C Michael White et al. (2002) [33], and Eugene Crystal et al. (2003) [35] included two amiodarone intervention groups, designated as 300 mg loading dose and 150 mg loading dose, slow load and fast load, and active use and discontinued use, respectively. Given that these 3 studies were each recorded as 2 comparisons, they were subsequently described as 40 studies and 43 comparisons in the following presentation. Despite the use of both fast and slow amiodarone loading strategies in the study by Satyendra Giri et al. (2001) [29], the absence of separate outcome reporting for these regimens led to their classification as a single comparison.

Administration of amiodarone was initiated preoperatively in 2 comparisons [33, 41], from preoperatively through postoperatively in 14 comparisons [23, 25, 27, 29, 31, 39, 40, 42, 50, 51, 60], intraoperatively in 6 comparisons [43, 53, 55, 56, 58, 59], intraoperatively to postoperatively in 5 groups [22, 26, 54, 57], and postoperatively in 15 comparisons [21, 24, 28, 30, 32, 34, 36–38, 44–46, 48, 49, 52]. And one comparison group lacked description regarding the timing of administration initiation [47]. Amiodarone was administered orally in 13 comparisons [23, 25, 29, 31, 33–35, 40, 41, 50, 60], intravenously in 12 comparisons [21, 24, 26–28, 43, 49, 53–55, 57], and via a combination of oral and intravenous routes in 15 comparisons [22, 30, 32, 36–39, 42, 44–48, 51, 52]. Additionally, using a polymer-based hydrogel on the epicardium was employed in 3 comparisons [56, 58, 59]. Due to missing specific parameters, the total amiodarone dose could not be calculated for 19 comparisons [26, 27, 30, 32, 35, 37, 40, 43, 45, 47, 49, 51, 56–60], and a low-dose regimen was administered in 5 groups [31, 41, 52, 53, 55], a medium-dose in 12 groups [22–25, 28, 29, 33, 34, 42, 44, 54], a high-dose regimen in 5 groups [33, 39, 46, 48, 50], and a very high-dose in 2 groups [21, 38].

### Risk of bias

**Fig. 2:**
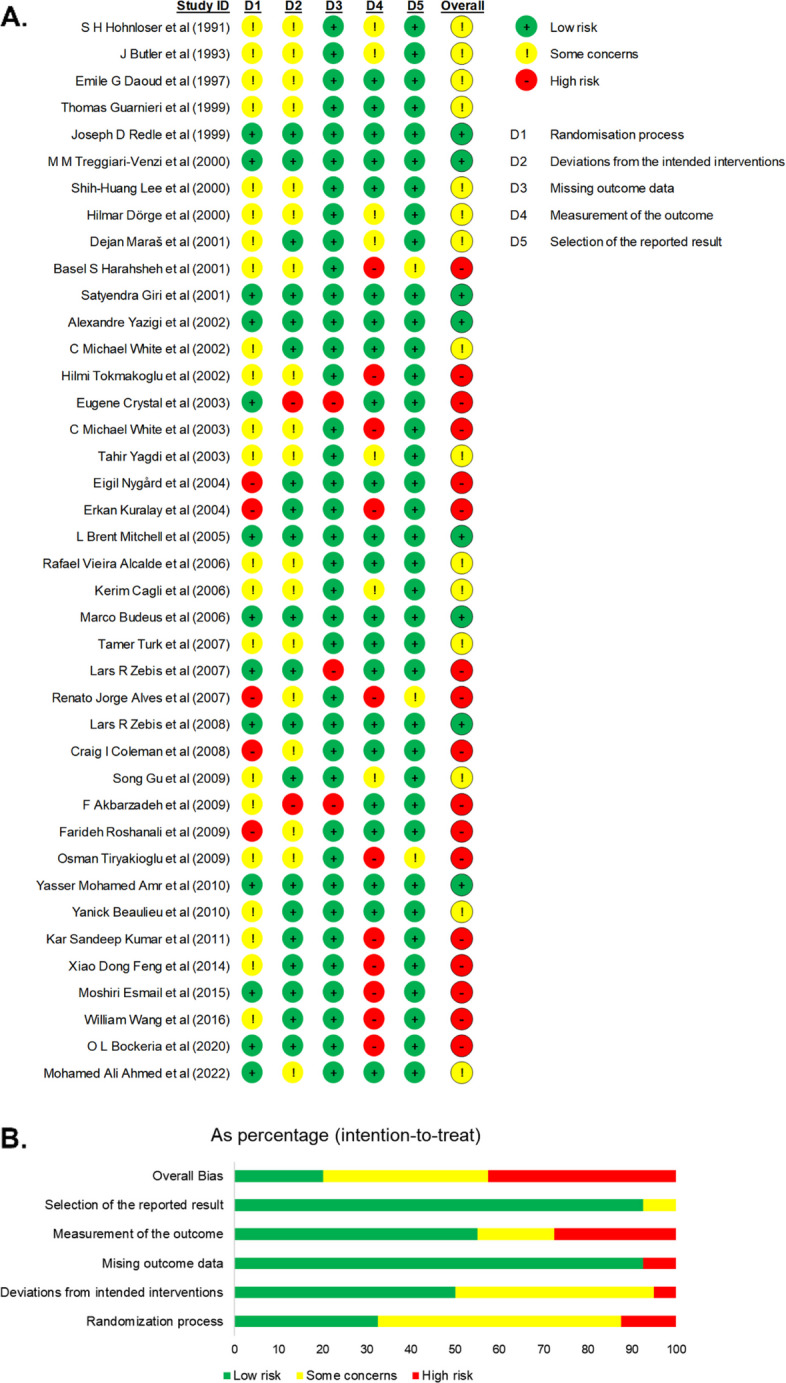
Risk of bias for included studies^1^

The risk of bias graph demonstrates the assessment for each dimension shown in Fig. 2. The assessment of the overall risk of bias yielded the following results: 8 studies (20.0%) were at low risk [25, 28, 29, 34, 40, 42, 48, 53], 17 studies (42.5%) were at high risk [30, 32, 35, 36, 38, 39, 44, 46, 47, 49, 51, 52, 55–59], and 15 studies (37.5%) were identified as having some concerns [21–24, 26, 27, 31, 33, 37, 41, 43, 45, 50, 54, 60].

Within the ROB-2 tool’s 5 domains, the ‘measurement of the outcome’ (11 studies, 27.5%) dimension was the main source of ‘high risk’ [30, 32, 36, 38, 44, 52, 55–59], due to these studies’ failure to report the specific POAF definition, including the AF duration threshold and time frame of onset, and detailed ECG monitoring protocols. The ‘some concerns’ rating was primarily identified in the ‘randomization process’ (22 studies, 55%) [21–24, 26, 27, 30–33, 36, 37, 41, 43, 45, 49, 50, 52, 54–56, 58] and ‘deviations from intended interventions’ domains (18 studies, 45%) [21–24, 26, 27, 30, 32, 36, 37, 41, 43–45, 47, 51, 52, 60], stemming from the lack of proper randomization or concealment and the absence of strict blinding, thereby potentially making researchers aware of patient group assignments.

**Fig. 3:**
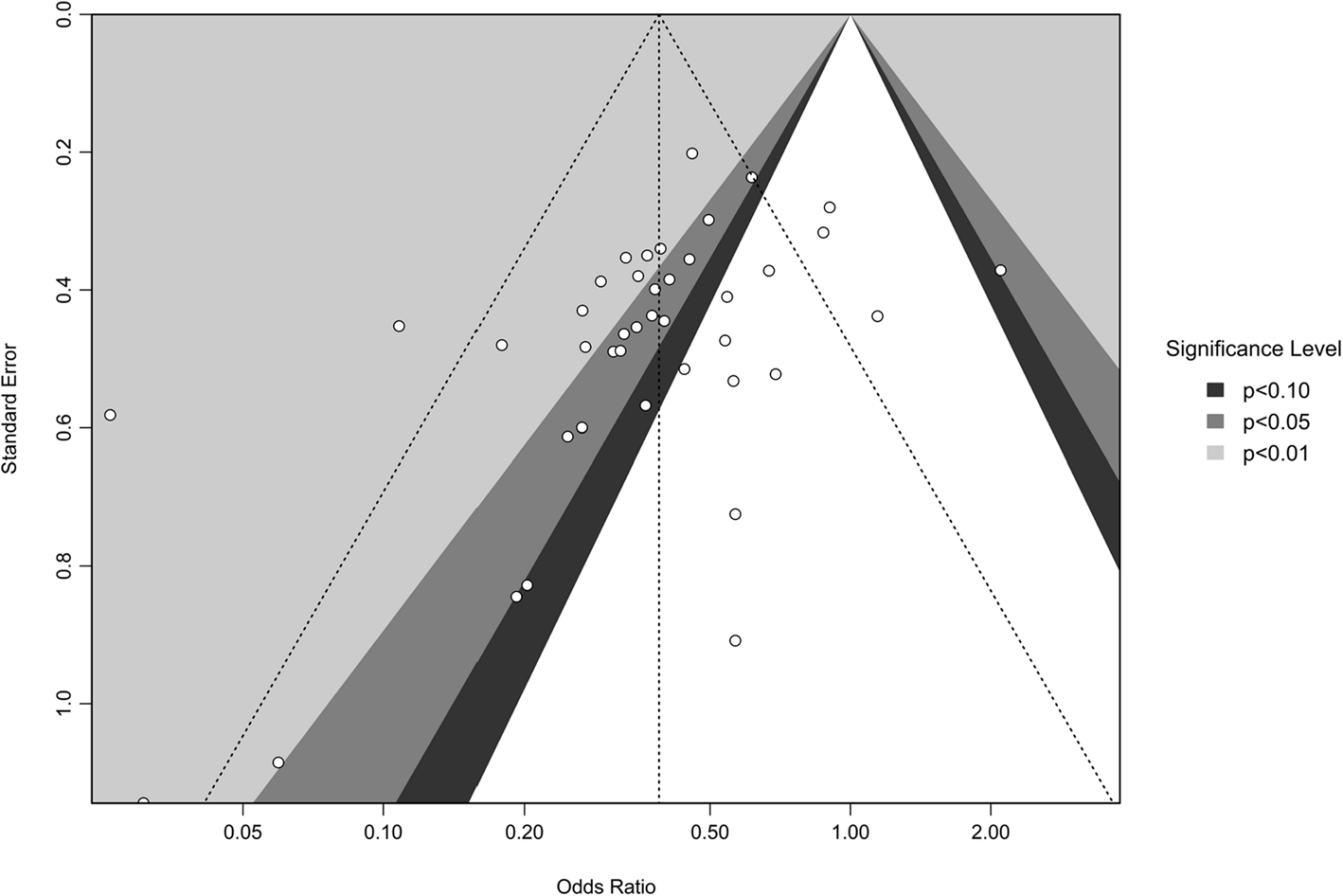
Funnel plot of comparisons

The contour-enhanced funnel plot demonstrated an asymmetric distribution of effect estimates (Fig. 3). The presence of funnel plot asymmetry alone does not allow definitive attribution to publication bias. However, consistent results were observed across Egger's test (t = −3.267; *P* = 0.002), Harbord's test (t = −1.950; *P* = 0.058), and Peter's test (t = −2.155; *P* = 0.037).

### Primary outcomes

**Fig. 4:**
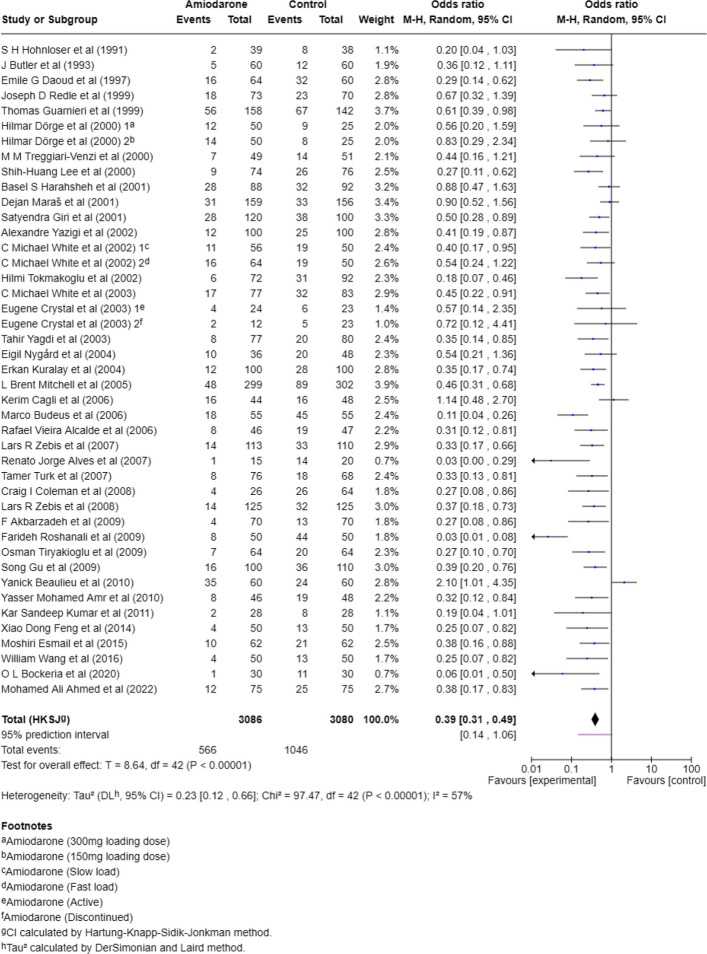
Forest plot: postoperative atrial fibrillation

The pooled results for the primary endpoint of POAF were shown in Fig. 4, which demonstrated that the amiodarone group had a significantly lower risk of POAF compared to the control group (OR = 0.39, 95% CI: 0.31 to 0.49; *P* < 0.00001), corresponding to a relative risk reduction of 61%. Substantial heterogeneity was observed among the studies (I^2^ = 57%). The 95% prediction interval (PI) ranged from 0.14 to 1.06.

### Subgroup

**Fig. 5:**
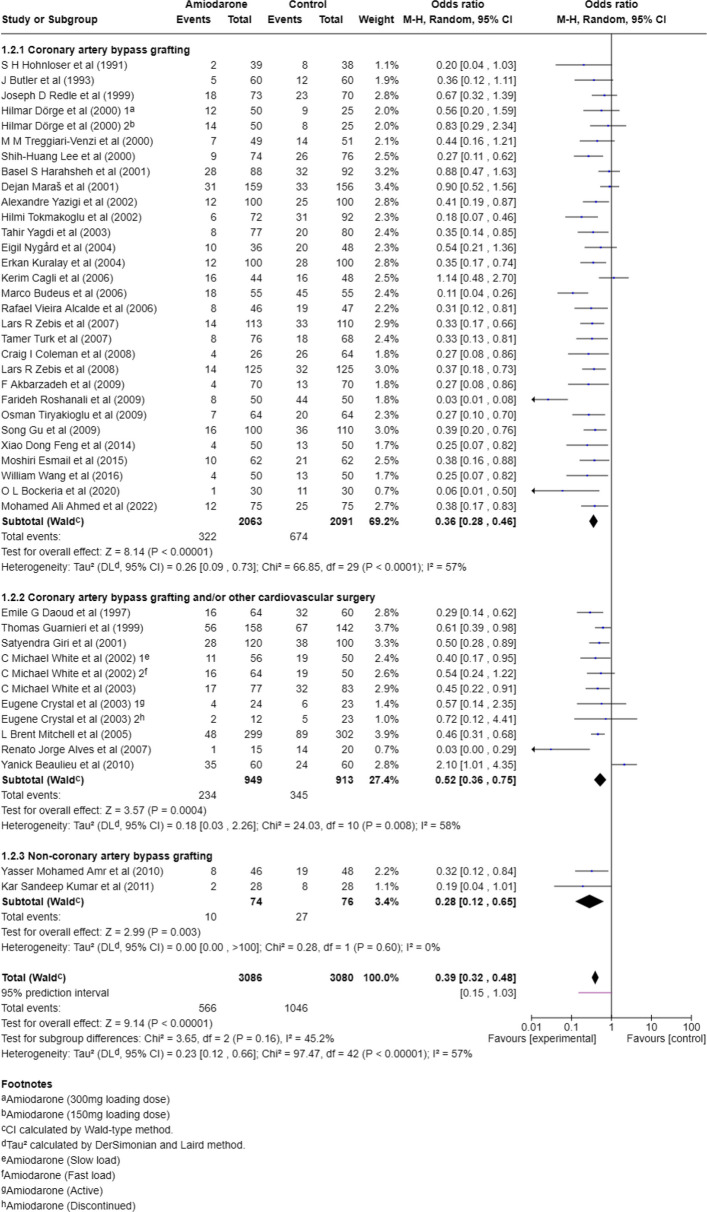
Forest plot: subgroup analysis of surgery type

Subgroup analysis by type of cardiac surgery demonstrated differences in effect estimates across surgical categories (Fig. 5). For patients undergoing isolated CABG, amiodarone demonstrated a significant preventive effect (OR = 0.36, 95% CI: 0.28 to 0.46; *P* < 0.00001), with substantial heterogeneity observed within this subgroup (*I*^2^ = 57%). A significant effect was also observed in studies that included CABG and/or other cardiac surgeries (OR = 0.52, 95% CI: 0.36 to 0.75; *P* = 0.0004), with heterogeneity (*I*^2^ = 58%). Only 2 studies evaluated patients undergoing non-CABG surgeries in which no statistically significant effect was observed (OR = 0.28, 95% CI: 0.12 to 0.65; *P* = 0.003), and heterogeneity was low (*I*^2^ = 0%).

**Fig. 6:**
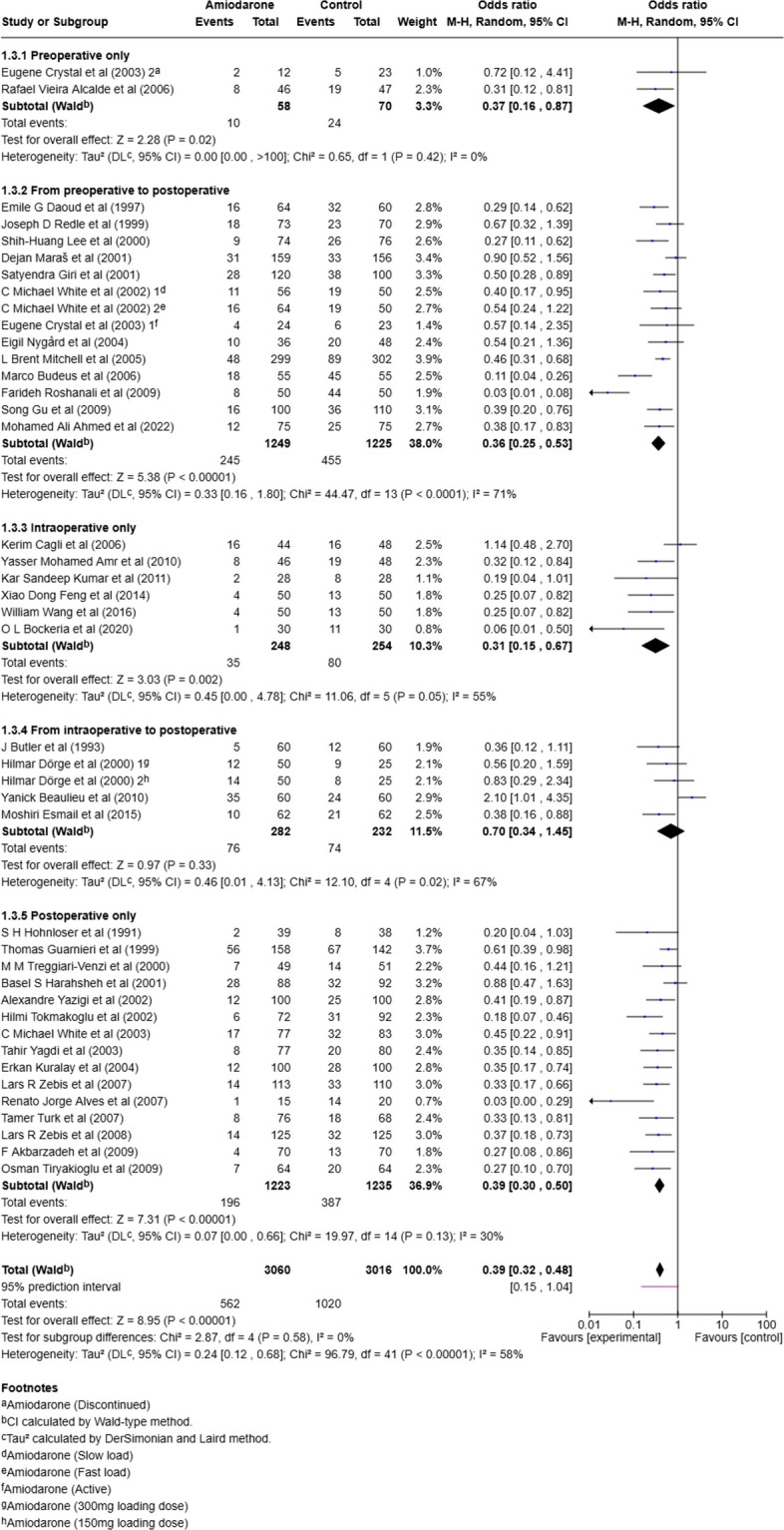
Forest plot: subgroup analysis of administration time

Significant reductions were observed in the preoperative-only period (OR = 0.37, 95% CI: 0.16 to 0.87; *P* = 0.02; *I*^2^ = 0%), from the preoperative to postoperative period (OR = 0.36, 95% CI: 0.25 to 0.53; *P* < 0.00001; *I*^2^ = 71%), the intraoperative-only period (OR = 0.31, 95% CI: 0.15 to 0.67; *P* = 0.002, *I*^2^ = 55%), and the postoperative-only period (OR = 0.39, 95% CI: 0.30 to 0.50; *P* < 0.00001; *I*^2^ = 30%) (Fig. 6). No statistically significant effect was observed in the from intraoperative to postoperative period (OR = 0.70, 95% CI: 0.34 to 1.45; *P* = 0.33; *I*^2^ = 67%).

**Fig. 7:**
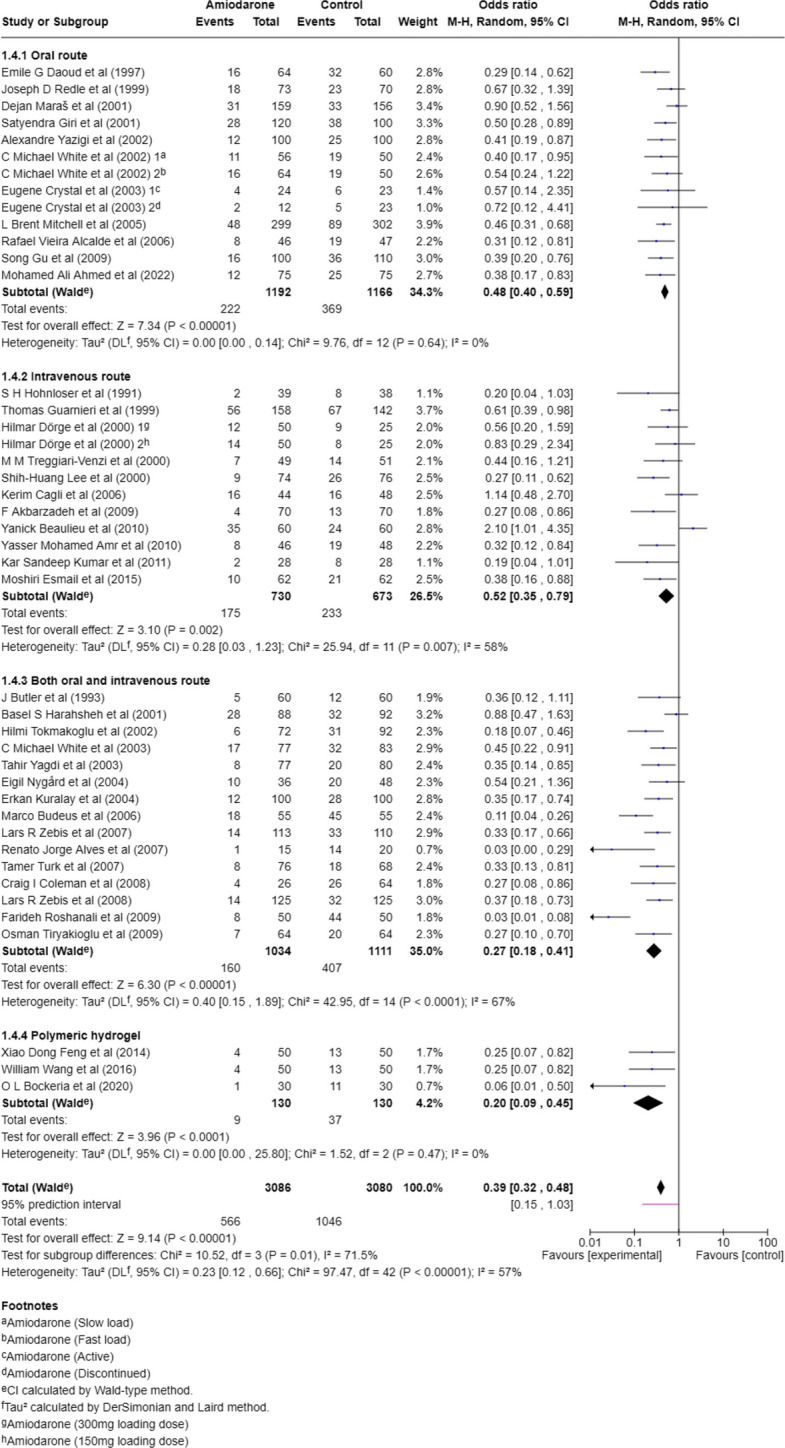
Forest plot: subgroup analysis of administration route

Subgroup analysis by route of administration revealed that significant efficacy was demonstrated with the oral route (OR = 0.48, 95% CI: 0.40 to 0.59; *P* < 0.00001; I^2^ = 0%), the intravenous route (OR = 0.52, 95% CI: 0.35 to 0.79; *P* = 0.002; I^2^ = 58%), and the both oral and intravenous route (OR = 0.27, 95% CI: 0.18 to 0.41; *P* < 0.00001; I^2^ = 67%), and hydrogel material (OR = 0.20, 95% CI: 0.09 to 0.45; *P* < 0.0001; I^2^ = 0%) (Fig. 7).

**Fig. 8:**
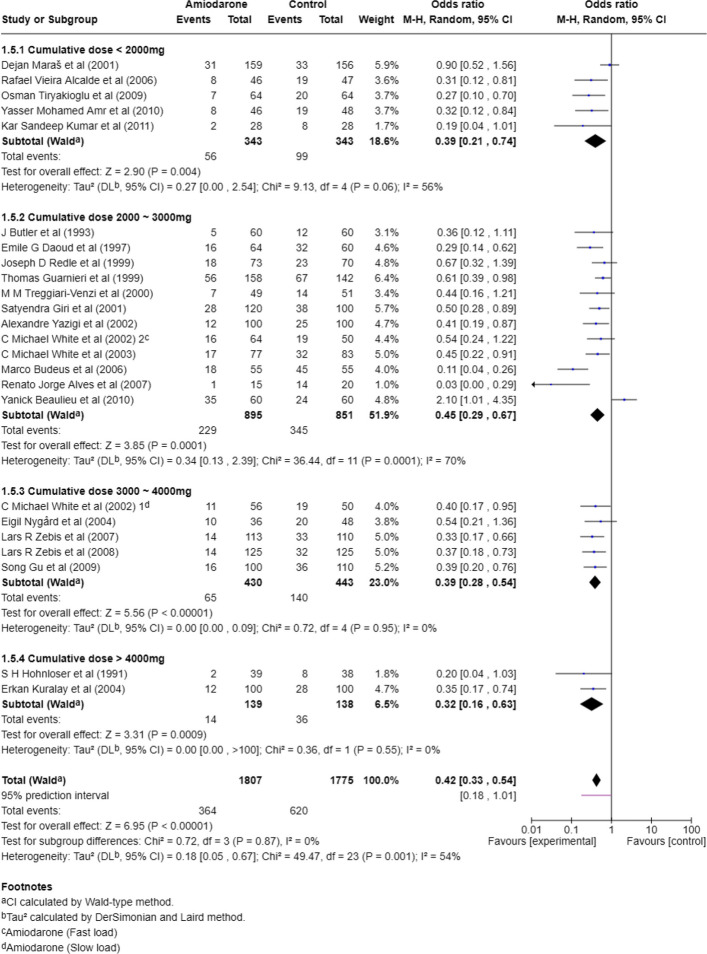
Forest plot: subgroup analysis of administration dose

Subgroup analysis by dose of administration showed that significant benefits in low-dose (OR = 0.39, 95% CI: 0.21 to 0.74; *P* = 0.004; I^2^ = 56%), middle-dose (OR = 0.45, 95% CI: 0.29 to 0.67; *P* = 0.0001; I^2^ = 70%), high-dose (OR = 0.39, 95% CI: 0.28 to 0.54; *P* < 0.00001; I^2^ = 0%), and very high (OR = 0.32, 95% CI: 0.16 to 0.63; *P* = 0.0009; I^2^ = 0%) subgroups (Fig. 8). Nineteen groups were excluded from the dose-group analysis due to unavailable dosing information and were categorized as ‘dose not available.’

### Sensitivity

Forest plots for sensitivity analysis were presented in Supplementary Appendix 2. Leave-one-out sensitivity analysis showed that the pooled effect estimates remained stable after sequentially removing each study (Supplementary Fig. 1). The recalculated pooled ORs ranged from 0.38 to 0.42 (the main analysis result was OR = 0.39). All recalculated 95% CIs did not cross the line of null effect (OR = 1) and largely overlapped with the overall 95% CI (0.31 to 0.50).

The pooled effect estimate using a fixed-effects model was consistent with the results from the random-effects model (Supplementary Fig. 2). The pooled OR under the random-effects model was 0.39 (95% CI 0.31 to 0.49; *P* < 0.00001), and the fixed-effects model produced a pooled OR of 0.42 (95% CI: 0.37 to 0.48; *P* < 0.00001).

When the analysis was restricted to the 23 studies assessed as having a ‘low-to-moderate risk of bias’ (Supplementary Fig. 3) [21–29, 31, 33, 34, 37, 40–43, 45, 46, 50, 53, 54, 60], the pooled effect estimate remained significant (OR = 0.47, 95% CI: 0.37 to 0.59; *P* < 0.00001). In this condition, heterogeneity was moderate (I^2^ = 50%). And the 95% PI ranged from 0.21 to 1.05, still intersecting with the line of null effect (OR = 1).

In analyses restricted to studies reporting POAF as the sole outcome, the pooled results were significant (OR = 0.39, 95% CI: 0.30 to 0.50; *P* < 0.00001) (Supplementary Fig. 4). In analyses restricted to studies with a completely defined diagnosis of POAF, the preventive effect of amiodarone remained statistically significantly associated with a reduced risk of POAF (OR = 0.41, 95% CI: 0.30 to 0.56; *P* < 0.00001) (Supplementary Fig. 5) [23, 25–27, 29, 31, 33–37, 39, 41–43, 45–47, 50, 51, 53, 54, 59, 60]; with the 95% PI crossing the null effect line (OR = 1).

A consistent result was observed when the analysis was restricted to studies employing continuous ECG monitoring (OR = 0.39, 95% CI: 0.31 to 0.50; *P* < 0.00001), with a 95% PI of 0.14 to 1.09 (Supplementary Fig. 6) [21–29, 31–34, 36–43, 45, 46, 48–51, 53, 54, 56, 58–60].

### Certainty of the evidence

The certainty of evidence for the primary outcome, including all 40 studies, was assessed using the GRADE approach. Although the initial certainty was high, as all included studies were RCTs, the overall certainty of evidence was downgraded to very low after assessment. Downgrading was applied due to concerns regarding risk of bias in some studies, substantial heterogeneity (I^2^ = 57%), and potential publication bias identified by the funnel plot. A GRADE assessment was performed for the studies included in the sensitivity analysis that were at low risk of bias or with some concerns (23 studies), using the same methodology. The evidence was rated as moderate, an upgrade attributable to the absence of downgrading for risk of bias and publication bias, unlike the assessment based on all 40 studies. Details of the GRADE assessment are provided in Supplementary Appendix 3.

### Meta-regression discovery

The study by Erkan Kuralay et al. [38] was excluded from the meta-regression analysis due to the administration duration, which extended for at least 4 months postoperatively and far exceeded the monitoring period. A total of 23 studies were included in the meta-regression analyses to examine the association between the cumulative dose of amiodarone (per mg) and its preventive effect [21–25, 28, 29, 31, 33, 34, 36, 39, 41, 42, 44, 46, 48, 50, 52–55].

**Fig. 9:**
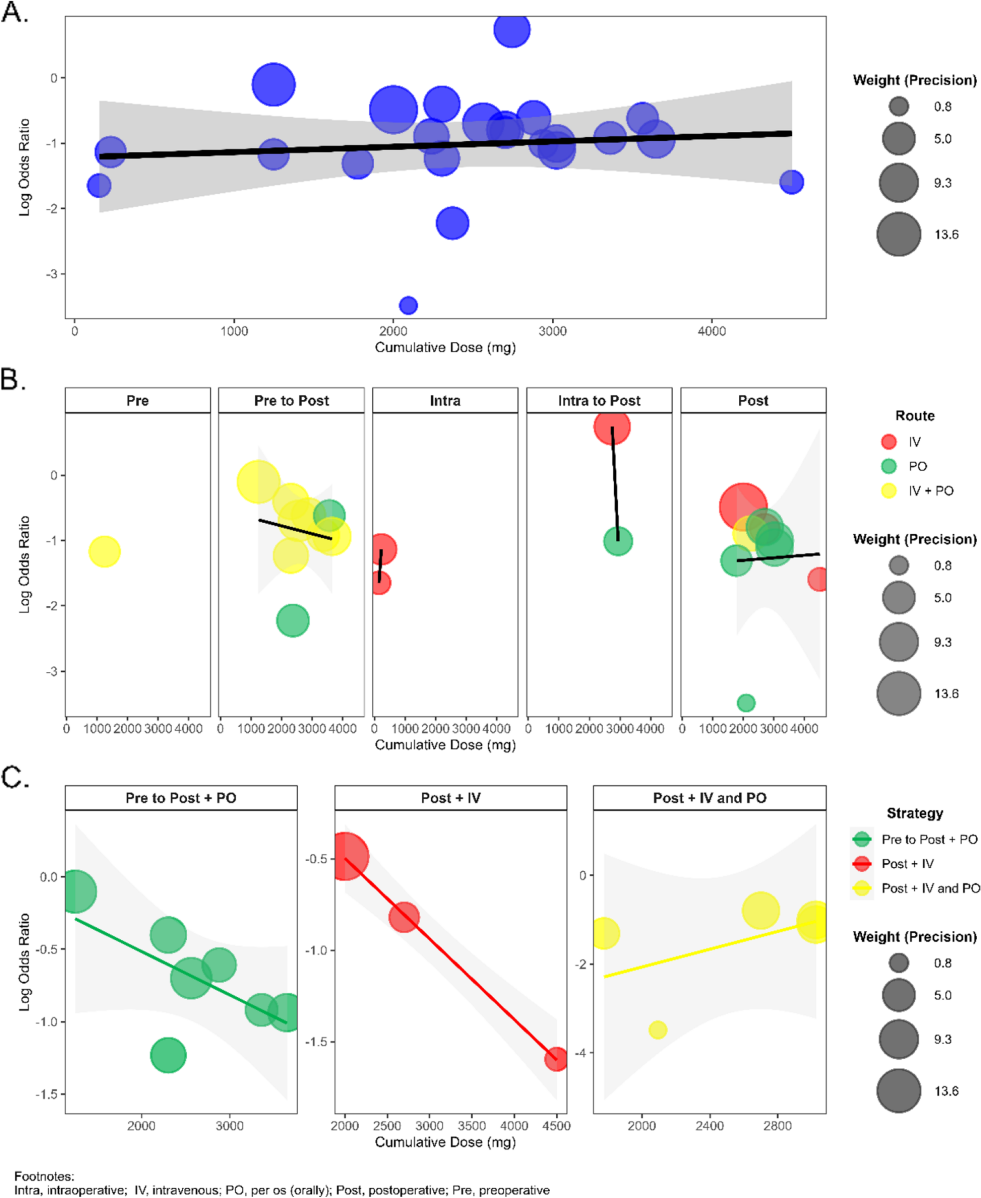
Bubble plot: univariable and multivariable meta-regression^2^

A univariable meta-regression analysis (K = 23, REML estimation) evaluated the relationship between cumulative amiodarone dose (per mg) and its preventive effect (Table 4, Fig. 9A). The overall test for the moderator was not significant (QM [df = 1] = 0.0422; *P* = 0.8372). Specifically, the dose coefficient was not statistically significant (β = 0.0001, 95% CI: −0.0003 to 0.0003; *P* = 0.8372). Significant heterogeneity was observed (QE [df = 21] = 48.6880; *P* = 0.0006; Tau^2^ = 0.2092; I^2^ = 56.53%).

**Table 4:** Univariate meta-regression analysis of amiodarone dose and its effect on postoperative atrial fibrillation prevention

Variable	Estimate (β)	Standard Error (SE)	95% confidence interval (CI)	*P*-value	Odds Ratio (OR) (per 1000 mg)	95% CI (per 1000 mg)
Intercept	−0.9349	0.3881	(−1.6955, −0.1743)	0.0160		
Dose (per mg)	0.0000	0.0001	(−0.0003, 0.0003)	0.8372	1.0312	(0.7692, 1.3825)

Model Information: Number of Studies (K) = 23; Tau^2^ = 0.2092; I^2^ = 56.53%; R^2^ = 0.00%. Test of Moderators: QM [df = 1] = 0.0422, *P* = 0.8372. Test for Residual Heterogeneity: QE [df = 21] = 48.6880, *P* = 0.0006

β, regression coefficient, indicates the mean change in the log (effect size) per unit increase in dose

*Abbreviations*: *Intra* Intraoperative, *IV* Intravenous, *PO* Per os (orally), *Post* Postoperative, *Pre* Preoperative

A multivariable meta-regression analysis (K = 23, REML estimation) included amiodarone dose (per mg), timing, and route as moderators (Table 5, Fig. 9B). The overall model test of moderators was statistically significant (QM [df = 7] = 17.1207; *P* = 0.0166). No significant heterogeneity was present in this model (QE [df = 15] = 23.6170; *P* = 0.0719; Tau^2^ = 0.0638; I^2^ = 27.41%). Compared to the univariable model, the multivariable model demonstrated a substantial improvement in explaining heterogeneity (R^2^ increased from 0.00% to 66.63%, and I^2^ decreased from 56.53% to 27.41%). Specifically, cumulative dose (per mg) remained non-significant (β = −0.0001, 95% CI: −0.0005 to 0.0002; *P* = 0.4048). Using postoperative administration as the reference category, the combined intraoperative-postoperative period was associated with a significant difference (β = 0.9704, 95% CI: 0.1574 to 1.7834; *P* = 0.0193), while other timings showed no significant difference, respectively, intraoperative (β = −1.0426; *P* = 0.1094), preoperative (β = −0.6510; *P* = 0.3249), or the combined preoperative-postoperative period (β = 0.0039; *P* = 0.9904). With intravenous administration as the reference, combined oral-intravenous administration showed a significant difference (β = −0.6830, 95% CI: −1.3127 to −0.0533; *P* = 0.0335), whereas oral-only administration did not differ significantly (β = −0.1391, 95% CI: −0.9505 to 0.6724; *P* = 0.7370).

**Table 5:** Multivariate meta-regression analysis of dose, timing, route, and their effect on postoperative atrial fibrillation prevention

Variable	Category/Reference	Estimate (β)	Standard Error (SE)	95% confidence interval (CI)	*P*-value	Odds Ratio (OR) (per 1000 mg)	95% CI (per 1000 mg)
Intercept		−0.2082	0.4823	(−1.1535, 0.7371)	0.6659		
Dose (per mg)	Continuous	−0.0001	0.0002	(−0.0005, 0.0002)	0.4048	0.8712	(0.6298, 1.2051)
Timing of Administration	Reference: Post						
	Intra	−1.0426	0.6512	(−2.3188, 0.2337)	0.1094		
	Intra to post	0.9704	0.4148	(0.1574, 1.7834)	0.0193		
	Pre	−0.6510	0.6613	(−1.9472, 0.6452)	0.3249		
	Pre to post	0.0039	0.3262	(−0.6355, 0.6433)	0.9904		
Route of Administration	Reference: IV						
	IV and PO	−0.6830	0.3213	(−1.3127, −0.0533)	0.0335		
	PO	−0.1391	0.4140	(−0.9505, 0.6724)	0.7370		

Model Information: Number of Studies (K) = 23; Tau^2^ = 0.0638; *I*^2^ = 27.41%; *R*^2^ = 66.63%. Test of Moderators: QM [df = 7] = 17.1207, *P* = 0.0166. Test for Residual Heterogeneity: QE [df = 15] = 23.6170, *P* = 0.0719

*Abbreviations*: *Intra* Intraoperative, *IV* Intravenous, *PO* Per os (orally), *Post* Postoperative, *Pre* Preoperative

To explore differences in dose–response relationships across specific strategies, subgroup meta-regression analyses were conducted (Table 6, Fig. 9C). In the “preoperative-postoperative oral” subgroup (K = 7), a statistically significant association between cumulative dose and effect size was observed (β = −0.0003; *P* = 0.0422; QE [df = 5] = 3.5003; *P* = 0.6233; I^2^ = 0%). Equivalent to each 1,000 mg increment in amiodarone dose, the incidence of POAF was reduced by 28.56% (OR = 0.7144, 95% CI: 0.5164 to 0.9882). In the “postoperative intravenous” subgroup (K = 3), no significant association between cumulative dose (per mg) and its preventive effect was identified (β = −0.0004; *P* = 0.1733; QE [df = 1] = 0.0015; *P* = 0.9696; I^2^ = 0%). Similarly, in the “postoperative oral combined intravenous” subgroup (K = 5), the dose effect was not statistically significant (β = 0.0004; *P* = 0.3634; QE [df = 3] = 4.5159; *P* = 0.2109; I^2^ = 0%).

**Table 6:** Subgroup meta-regression analysis of the dose–response relationship by key administration strategies

Subgroup (Timing + Route)	Dose Estimate (β)	Standard Error (SE)	95% confidence interval (CI)	*P*-value	Odds Ratio (OR) (per 1000 mg)	95% CI (per 1000 mg)
Pre to Post + PO^a^	−0.0003	0.0002	(−0.0007, −0.0000)	0.0422	0.7144	(0.5164, 0.9882)
Post + IV^b^	−0.0004	0.0003	(−0.0011, 0.0002)	0.1733	0.6392	(0.3356, 1.2173)
Post + PO and IV^c^	0.0004	0.0004	(−0.0004, 0.0012)	0.3634	1.4674	(0.6418, 3.3550)

The combinations "Intra to Post" and "IV and PO," which were significant in the multivariate analysis, did not show a significant dose effect within their specific subgroups, likely due to limited study numbers or different combined categories

*Abbreviations*: *Intra* Intraoperative, *IV* Intravenous, *PO* Per os (orally), *Post* Postoperative, *Pre* Preoperative

^a^Model Information: Number of Studies (K) = 7; Tau^2^ = 0; *I*^2^ = 0.00%; *R*^2^ = 100.00%. Test of Moderators: QM [df = 1] = 4.1280, *P* = 0.0422. Test for Residual Heterogeneity: QE [df = 5] = 3.5003, *P* = 0.6233

^b^Model Information: Number of Studies (K) = 3; Tau^2^ = 0; *I*^2^ = 0.00%; *R*^2^ = 0.00%. Test of Moderators: QM [df = 1] = 1.8546, *P* = 0.1733. Test for Residual Heterogeneity: QE [df = 1] = 0.0015, *P* = 0.9696

^c^Model Information: Number of Studies (K) = 5; Tau^2^ = 0; *I*^2^ = 0.00%; *R*^2^ = 0.00%. Test of Moderators: QM [df = 1] = 0.8263, *P* = 0.3634. Test for Residual Heterogeneity: QE [df = 3] = 4.5159, *P* = 0.2109

### Secondary outcomes

Forest plots for secondary outcomes are presented in Supplementary Appendix 2. Patients receiving amiodarone had a significantly shorter HLOS (MD = −1.33, 95% CI −1.89 to −0.77; *P* < 0.0001; I^2^ = 93%) (based on 25 comparison groups, Supplementary Fig. 7) [21–24, 26, 27, 29, 31, 33, 34, 36–42, 46, 50, 51, 53, 54, 59, 60] and lower incidence of CVA (based on 20 comparison groups, Supplementary Fig. 8) (OR = 0.59, 95% CI: 0.36 to 0.96; *P* = 0.04; I^2^ = 0%) [22–24, 27, 29, 31, 33, 34, 36–38, 40, 41, 45, 46, 53, 54, 56, 58]. Amiodarone was also associated with a higher risk of bradycardia (OR = 2.33, 95% CI: 1.67 to 3.26; *P* < 0.00001; I^2^ = 0%) (based on 23 comparison groups, Supplementary Fig. 9) [21, 22, 25, 26, 29, 31, 34, 36, 37, 40, 42, 43, 45–47, 50, 51, 53, 55, 56, 58, 60] than controls.

No statistically significant difference was observed in ILOS (MD = −0.36, 95% CI: −0.78 to 0.05; *P* = 0.08; I^2^ = 88%) (based on 16 comparison groups, Supplementary Fig. 10) [27–29, 31, 33, 35, 37, 38, 42, 51, 53–55, 60]. The pooled estimate showed no significant effect between amiodarone use and CVM (OR = 1.69, 95% CI 0.73 to 3.89; *P* = 0.19; I^2^ = 1%) (based on 9 comparison groups, Supplementary Fig. 11) [23–25, 27, 28, 31, 42, 45, 49] or ACM after cardiac surgery (OR = 1.05, 95% CI: 0.74 to 1.50; *P* = 0.78; I^2^ = 0%) (based on 26 comparison groups, Supplementary Fig. 12) [22–29, 31, 33, 35–37, 39, 40, 42, 45, 46, 49, 50, 53, 54]. No significant differences were observed in heart block (OR = 0.86, 95% CI: 0.38 to 1.91; *P* = 0.70; I^2^ = 0%) (based on 10 comparison groups, Supplementary Fig. 13) [22, 29, 31–34, 38, 42, 50] or hypotension (OR = 1.26, 95% CI 0.87 to 1.83; *P* = 0.22; I^2^ = 0%) (including 15 comparison groups, Supplementary Fig. 14) [29, 33, 36, 37, 40, 42, 43, 46, 47, 50, 51, 53, 55, 57] occurrence between the amiodarone and control groups.

## Discussion

### Main findings

This systematic review and meta-analysis of 40 RCTs involving 6,166 patients suggests that perioperative prophylactic amiodarone may be associated with a lower risk of POAF in cardiac surgery patients, resulting in an approximate 61% reduction. In addition, prophylactic amiodarone use may be associated with a shorter HLOS and a lower incidence of CVA, at the expense of an increased risk of bradycardia.

Nevertheless, the overall certainty of evidence was rated as very low according to the GRADE framework, due to substantial heterogeneity among the included studies, a considerable proportion of studies with risk of bias, and potential publication bias. Furthermore, the 95% PI for the pooled estimates, across the primary analysis and multiple sensitivity analyses, consistently crossed the line of null effect (OR = 1), indicating that the effect of amiodarone may vary across different settings. Therefore, these findings should be interpreted as hypothesis-generating rather than conclusive, suggesting that amiodarone may represent a potentially beneficial preventive strategy for POAF, while the true effect size remains uncertain to some extent.

### Interpretation of the results

The present analysis suggests that prophylactic amiodarone is associated with a reduced incidence of POAF after cardiac surgery, consistent with previous meta-analyses and current international guidelines (2024 ESC/EACTS and 2023 ACC/AHA guidelines) [9, 10, 19, 61, 62]. However, the prophylactic effect should be interpreted in the context of substantial between-study variability. The substantial heterogeneity reflects the considerable clinical and methodological diversity across trials, including surgical populations, administration timing, routes of delivery, dosing strategies, outcome definitions, and ECG monitoring methods. Importantly, the consistent crossing of the null line by the 95% PI indicates that the magnitude, and even the presence, of benefit may not be consistent across clinical settings. It would be valuable to conduct thorough subgroup and sensitivity analyses to gain further insight.

Subgroup analyses explored sources of this heterogeneity, suggesting that the effectiveness of amiodarone is strongly influenced by the combination of administration timing and route rather than cumulative dose alone. Notably, in view of the methodological heterogeneity and evidence limitations, these results should be interpreted with caution and considered hypothesis-generating.

Across analyses, both the preoperative through postoperative period and the postoperative period demonstrated comparable and significant efficacy. However, regimens of postoperative initiation, which may be more feasible in clinical practice, showed lower heterogeneity, implying that a prophylactic strategy starting after the surgical injury may be more consistently effective across different settings. In contrast, regimens initiated intraoperatively, with or without postoperative continuation, demonstrated more variable and less predictable effects, possibly reflecting the influence of anesthesia and CPB on drug distribution and metabolism.

Regarding the route of administration, the combined oral and intravenous administration appeared more effective than either route alone, likely attributable to its facilitation of both rapid achievement and sustained maintenance of therapeutic serum and tissue concentrations. While the effect sizes for single-route (oral or intravenous) administration were weaker, both routes appeared to be effective. Furthermore, while local epicardial hydrogel delivery appears to be an effective innovative route, as only three studies utilized this method, these findings require further validation.

No clear dose–response relationship was observed in the simple dose-stratified analysis, indicating that merely increasing cumulative dose does not reliably enhance prophylactic efficacy. This observation was supported by univariable meta-regression, in which, ignoring the timing and route of administration, cumulative dose alone failed to explain any of the inter-study heterogeneity. Only when dose, timing, and route were modeled simultaneously did heterogeneity substantially decrease, indicating that dosing effects are context-dependent rather than universal. These findings were consistent with the results of previous subgroup analysis, strongly suggesting that the variation in efficacy and heterogeneity observed clinically was primarily resulting from different combinations of administration timing and route, rather than dose differences alone.

Further subgroup meta-regression supports this interpretation. A significant dose–response relationship was identified only within the specific strategy of oral administration from the preoperative to postoperative period, and within this strategy, higher cumulative doses may be associated with a superior efficacy, whereas no dose effect was observed in postoperative intravenous or combined regimens. These findings suggest that dose optimization may be meaningful only within selected administration protocols and that clinical effectiveness is primarily driven by how and when amiodarone is delivered rather than by simply increasing the cumulative dose.

Finally, although amiodarone use was associated with an increased risk of bradycardia, consistent with its pharmacological profile, no corresponding differences were observed in mortality, heart block, or hypotension. A shorter HLOS was observed; despite substantial heterogeneity and a 93% PI encompassing the null line, the finding remained statistically significant in a leave-one-out sensitivity analysis. In addition, a modest reduction in CVA was also noted; however, this finding should be interpreted cautiously. Although the overall heterogeneity was low, the result lost its statistical significance in a leave-one-out sensitivity analysis when the studies by Dejan Maras et al., Satyendra Giri et al., C. Michael White et al. (2002, 2003), and L. Brent Mitchell et al. were individually removed.

### Comparison with previous studies

The meta-analysis by Kyle A Arsenault et al. [61], including 33 studies, demonstrated that amiodarone for POAF prevention yielded an OR of 0.43 (95% CI: 0.34 to 0.54; *P* < 0.00001) and reduced the HLOS (MD = −0.95, 95% CI: −1.37 to −0.52; *P* < 0.0001). However, no significant differences were observed in the endpoints of CVA (*P* = 0.06), CVM (*P* = 0.83), or ACM (*P* = 0.70). The results for amiodarone in preventing POAF exhibited heterogeneity (I^2^ = 63%), and the study did not perform subgroup analyses to explore the sources of this heterogeneity.

Mitchell S. Buckley et al. [19] included 14 studies and found that all three low (< 3,000 mg), medium (3,000 to 5,000 mg), and high (> 5,000 mg) dose groups of converted oral amiodarone reduced the incidence of POAF (OR = 0.58; OR = 0.45; OR = 0.44; respectively), with no significant differences observed between the groups (*P* = 0.238), although over 3,000 mg appeared to be associated with superior efficacy. When stratified by initiation timing of preoperative and postoperative, there were no significant differences between the two groups (OR = 0.50; OR = 0.48; *P* = 0.862). The authors also concluded that preoperative initiation of amiodarone may not be necessary.

Based on a pooled analysis of 23 studies, Saurav Chatterjee et al. [62] reported that both oral-only (risk ratio [RR] = 0.59; *P* < 0.01) and combined oral-intravenous (RR = 0.57; *P* < 0.01) regimens significantly decreased the risk of POAF, consistent with the findings of Mitchell S. Buckley et al. [19]. Besides, preoperative (RR = 0.55; *P* < 0.01) and postoperative (RR = 0.50; *P* = 0.0009) initiation demonstrated comparable effectiveness, and preoperative amiodarone lasting more than one day did not provide additional benefit (*P* = 0.98). Moreover, sensitivity analysis revealed a lower risk of POAF compared to the comparator group when at least a 300 mg loading dose and a total dose of 1 g were administered intravenously.

While the core findings of prior meta-analyses were broadly consistent with ours and the pooled results estimated comparable, earlier studies primarily focused on pooled efficacy estimates and simple dose stratifications, with limited exploration of the sources of heterogeneity. In contrast, our study provides important updates and refinements by systematically integrating timing, route, and dose within a unified analytical framework.

We utilized drug doses as reported in the original trials or calculated according to predefined methodologies, applying a unified bioavailability conversion method. No imputation was performed when key variables were unavailable. Although this approach reduced the number of studies eligible for dose-based analyses, it improved internal consistency and provided more comparable study-level assessments. In exploratory multivariable meta-regression analyses, the timing and route of administration explained a substantial proportion of the observed heterogeneity, whereas the cumulative dose alone may play only a secondary role.

Furthermore, the inclusion of more recent trials and novel epicardial hydrogel delivery strategies provided updated insight into evolving prophylactic approaches. Although these newer strategies appear promising, the limited number of studies and their substantial risk of bias preclude definitive conclusions, highlighting the need for further validation in more generalizable populations.

### Limitations

Several important limitations should be acknowledged. First, a substantial proportion of the included studies (42.5%) were judged to be at high risk of bias, primarily due to inadequacies in the randomization process and lack of blinding, which may have led to an overestimation of the true treatment effect.

Second, the asymmetry in the contour-enhanced funnel plot, together with significant Egger’s, Harbord’s, and Peter’s tests, indicated a potential for publication bias, wherein studies with negative results may remain unpublished.

Third, in addition to the heterogeneity introduced by administration regimens addressed in subgroup analyses, variability may have been amplified by inconsistent definitions of the primary outcome and differences in ECG monitoring intensity. Due to historical context, some of the earlier trials included in our analysis utilized composite endpoints, such as any AFL or SVT, rather than restricting the outcome to strictly defined POAF. Importantly, our methodology of including these studies in the primary analysis aligns with the precedent set by several previously published meta-analyses on this topic. Furthermore, we note that major clinical guidelines (AHA/ACC/ESC) for AF management, from 2001 to 2024, have not provided a consistently clear, operational definition for POAF. The evidence base cited within these guidelines often includes the very same meta-analyses and early RCTs that employ broader endpoint definitions, reflecting a longstanding ambiguity in the field. To address this heterogeneity, we performed pre-specified sensitivity analyses that pooled only those studies with the endpoint of POAF (excluding AFL or SVT) and clearly defined POAF. However, substantial statistical heterogeneity persisted even in those analyses. An examination of our Table 3 further illustrates that among studies ostensibly focused on POAF, the specific definitions and duration criteria varied considerably, which is likely a major contributor to the observed heterogeneity. In the absence of a universally accepted, precise qualitative and quantitative definition for POAF, and given the differences in detection methods across study centers, such heterogeneity is an inherent and expected limitation when synthesizing the existing studies in this area.

Fourth, the overall certainty of evidence for the primary outcome was rated as very low according to the GRADE framework. This downgrading was driven by signals of publication bias, the methodological limitations of the included trials, and substantial heterogeneity. Notably, in a sensitivity analysis limited to the 23 studies judged as low risk of bias or some concerns, the risk of bias and publication bias domains were rated as not serious in the GRADE assessment. This resulted in a moderate overall certainty of evidence, which is higher than the GRADE rating obtained when all 40 studies were included.

Fifth, meta-regression and subgroup analyses are observational by nature and susceptible to ecological bias. Consequently, findings regarding optimal timing, route, or dose should be interpreted as exploratory rather than definitive and should not be viewed as substitutes for adequately powered randomized comparisons. In addition, incomplete reporting of dosing parameters limited the ability to calculate cumulative dose in nearly half of the included comparisons, reducing statistical power for dose–response analyses.

Sixth, the subgroup analyses (Figs. 5, 6, 7 and 8) and meta-regression analyses (Fig. 9C and Table 6) were likely underpowered due to the limited number of studies in certain subgroups. This paucity of data increases the risk of both false-negative findings (masking true subgroup effects) and false-positive findings (due to chance) and precludes robust adjustment for potential confounders. Therefore, these exploratory results should be interpreted with caution.

Finally, the restriction to English-language publications introduces a potential language bias, which may have excluded relevant trials conducted and reported in other languages.

### Implications for clinical practice

It is important to acknowledge that surgical practice has evolved substantially since the publication of the earliest trials included in this meta-analysis. During this period, several significant advancements may have influenced the generalizability of our findings to contemporary practice.

First, surgical techniques have shifted toward less invasive approaches, including off-pump coronary artery bypass (OPCAB), minimally invasive direct coronary artery bypass (MIDCAB), robotic-assisted CABG, and minimally invasive cardiac surgery (MICS) CABG [63]. These techniques aim to reduce surgical trauma and may impact the risk of postoperative arrhythmia. Second, perioperative management has improved with the introduction of Enhanced Recovery After Surgery (ERAS) protocols in cardiac surgery since 2019, incorporating multimodal pain management, early extubation strategies, and goal-directed fluid therapy [64]. Third, advancements in CPB technology, including refined myocardial protection strategies and biocompatible circuit coatings, have potentially reduced myocardial ischemia/reperfusion injury and the occurrence of adverse events [65, 66]. Additionally, perioperative pharmacological management, including broader use of beta-blockers, statins, and angiotensin-converting enzyme inhibitors/angiotensin II receptor blockers, may have modified the baseline risk of complications in contemporary cohorts [13].

These temporal changes in surgical techniques and perioperative care should be considered when extrapolating our findings to current clinical practice, as the treatment effect observed in older trials may not fully reflect outcomes achievable with modern multidisciplinary care pathways.

In light of the limitations of the included studies and the low certainty of the current evidence, the subsequent discussion serves only to highlight the different pharmacological approaches identified in this review, which may inform the design of future trials. Rather than supporting a universal dosing target, the findings suggest that clinical effectiveness depends primarily on the combination strategy of administration timing and route, depending on the patient’s clinical context and available medical resources, rather than dwelling on a specific dose.

From a practical perspective, two general approaches appear reasonable. For elective surgeries in which preoperative strategy is feasible, oral administration initiated before surgery and continued postoperatively may allow dose individualization. In contrast, for urgent or unplanned surgeries, postoperative initiation, using the intravenous route alone or combined with the oral route, may provide some preventive effect.

Regardless of the chosen strategy, clinicians should be vigilant about the risk of bradycardia and implement appropriate monitoring. Importantly, the increased incidence of bradycardia observed in this analysis may not be associated with higher mortality or major conduction disturbances, suggesting that this adverse effect is generally manageable in appropriately monitored settings.

Given the very low certainty of evidence, these recommendations should be interpreted cautiously and viewed as possible reminders of clinical practice rather than guidance or prescriptive standards. High-quality, contemporary randomized trials using standardized POAF definitions and monitoring protocols are needed to confirm these findings and to more precisely define the optimal prophylactic strategy.

## Conclusion

This meta-analysis suggests that perioperative prophylactic amiodarone may be associated with a reduced incidence of POAF in patients undergoing cardiac surgery and may also confer modest benefits in selected secondary outcomes, including HLOS and CVA, at the expense of an increased risk of bradycardia. However, its effectiveness appears to depend on specific combinations of administration timing and route rather than cumulative dose alone. High-quality, contemporary randomized trials with standardized POAF definitions, rigorous monitoring protocols, and prespecified administration strategies are required to more precisely define the clinical role of prophylactic amiodarone in cardiac surgery.

## Supplementary Information

Supplementary Material 1.

Supplementary Material 2.

Supplementary Material 3.

## Data Availability

This study is a systematic review and meta-analysis. All data underlying this analysis were derived from the published studies included in this review. The derived dataset extracted and synthesized for this meta-analysis is available from the corresponding author upon reasonable request. Access to data may be subject to compliance with the original studies' data use agreements.

## Abbreviations

ACM: All-cause mortality
AF: Atrial fibrillation
AFL: Atrial flutter
AKI: Acute kidney injury
CABG: Coronary artery bypass grafting
CI: Confidence interval
CPB: Cardiopulmonary bypass
CVA: Cerebrovascular accident
CVM: Cardiovascular mortality
DL: DerSimonian and Laird
ECG: Electrocardiograph
FDA: Food and Drug Administration
GRADE: Grading of Recommendations Assessment, Development and Evaluation
HLOS: Hospital length of stay
HF: Heart failure
HKSJ: Hartung-Knapp-Sidik-Jonkman
ICU: Intensive care unit
ILOS: Intensive care unit length of stay
LOS: Length of stay
LVEF: Left ventricular ejection fraction
MD: Mean difference
MICS: Minimally invasive cardiac surgery
MIDCAB: Minimally invasive direct coronary artery bypass
OPCAB: Off-pump coronary artery bypass
OR: Odds ratio
PI: Prediction interval
POAF: Postoperative atrial fibrillation
PRISMA: Preferred Reporting Items for Systematic Reviews and Meta-Analysis Quality of Reporting of Meta-Analysis
RCTs: Randomized controlled trials
REML: Restricted maximum likelihood
ROB-2: Risk of bias 2
RR: Risk ratio
SVT: Supraventricular tachyarrhythmia
WT: Wald-type

## Footnotes

1 A. Risk of bias graph for each included study. B. Risk of bias summary for each included study.

2 A. Bubble plot of meta-regression: cumulative amiodarone dose (mg) vs. log OR for POAF. B. Bubble plot of meta-regression: cumulative amiodarone dose (mg) vs. log OR for POAF, including administration timing and route as moderators, with Post and IV serving as the reference levels. C. Bubble plot of meta-regression: cumulative dose (mg) vs. log OR for POAF, with three subgroups: (1)Pre to Post + PO; (2) Post + IV; (3) Post + PO and IV. Abbreviations: Intra, intraoperative; IV, intravenous; OR, odds ratio; PO, per os (orally); POAF, postoperative atrial fibrillation; Post, postoperative; Pre: preoperative.
