# Supplementary material for: Meta-analysis of perioperative amiodarone for prevention of postoperative atrial fibrillation (POAF) in cardiac surgery patients: update and reevaluation of timing, route, and dosage
Source: BMC Cardiovasc Disord. 2026 May 7;26:547. doi: 10.1186/s12872-026-05813-w (PMC13321565; doi:10.1186/s12872-026-05813-w)
Supplement: Supplementary file 1 — Supplementary Material 1. [file 12872_2026_5813_MOESM1_ESM.docx]

**Supplementary Appendix 1**

**PubMed**

(((((((((((((((((((((periprocedural) OR (peri-procedural)) OR (procedure-related)) OR (post-procedural)) OR (postprocedural)) OR (perioperative)) OR (intraoperative)) OR (postoperative)) OR (peri-operative)) OR (post-operative)) OR (perisurgical)) OR (postsurgical)) OR (peri-surgical)) OR (post-surgical)) OR (after surger)) OR (after cardiac surger)) OR ((postoperative period) OR (postoperative complications))) OR ("Coronary Artery Bypass"[Mesh])) OR ("Cardiac Surgical Procedures"[Mesh])) AND ((((((atrial fibrillation) OR (atrium fibrillation)) OR (auricular fibrilation)) OR (auricular fibrillation)) OR (AF)) OR ("Atrial Fibrillation"[Mesh]))) OR (POAF)) AND ((amiodarone) OR ("Amiodarone"[Mesh]))

**Embase**

((periprocedural OR 'peri procedural' OR 'procedure related' OR 'post procedural' OR postprocedural OR perioperative OR intraoperative OR postoperative OR 'peri operative' OR 'post operative' OR perisurgical OR postsurgical OR 'peri surgical' OR 'post surgical' OR (after AND surger) OR (after AND ('cardiac'/exp OR cardiac) AND surger) OR (postoperative AND period) OR (postoperative AND ('complications'/exp OR complications))) AND (atrial AND ('fibrillation'/exp OR fibrillation) OR (('atrium'/exp OR atrium) AND ('fibrillation'/exp OR fibrillation)) OR (auricular AND fibrilation) OR (auricular AND ('fibrillation'/exp OR fibrillation)) OR af) OR poaf) AND ('amiodarone'/exp OR amiodarone)

**The Cochrane Library**

((((((((((((((((periprocedural) OR (peri-procedural)) OR (procedure-related)) OR (post-procedural)) OR (postprocedural)) OR (perioperative)) OR (intraoperative)) OR (postoperative)) OR (peri-operative)) OR (post-operative)) OR (perisurgical)) OR (postsurgical)) OR (peri-surgical)) OR (post-surgical)) OR (after surger)) OR (after cardiac surger)):ti,ab,kw OR ((postoperative period) OR (postoperative complications)):ti,ab,kw AND (((((atrial fibrillation) OR (atrium fibrillation)) OR (auricular fibrilation)) OR (auricular fibrillation)) OR (AF)):ti,ab,kw OR (POAF):ti,ab,kw AND (amiodarone):ti,ab,kw
