# Supplementary material for: Meta-analysis of perioperative amiodarone for prevention of postoperative atrial fibrillation (POAF) in cardiac surgery patients: update and reevaluation of timing, route, and dosage
Source: BMC Cardiovasc Disord. 2026 May 7;26:547. doi: 10.1186/s12872-026-05813-w (PMC13321565; doi:10.1186/s12872-026-05813-w)
Supplement: Supplementary file 2 — Supplementary Material 2. [file 12872_2026_5813_MOESM2_ESM.docx]

**Supplementary Appendix 2**

**Content**

**Supplementary Figure 1. Forest plot: leave-one-out method**

**Supplementary Figure 2. Forest plot: fixed-effect model**

**Supplementary Figure 3. Forest plot: studies with low to moderate risk of bias**

**Supplementary Figure 4. Forest plot: studies reporting POAF as the sole outcome**

**Supplementary Figure 5. Forest plot: studies with a complete POAF definition**

**Supplementary Figure 6. Forest plot: studies with continuous ECG monitoring**

**Supplementary Figure 7. Forest plot: amiodarone prophylaxis and HLOS**

**Supplementary Figure 8. Forest plot: amiodarone prophylaxis and the risk of CVA**

**Supplementary Figure 9. Forest plot: amiodarone prophylaxis and the risk of bradycardia**

**Supplementary Figure 10. Forest plot: amiodarone prophylaxis and ILOS**

**Supplementary Figure 11. Forest plot: amiodarone prophylaxis and the risk of CVM**

**Supplementary Figure 12. Forest plot: amiodarone prophylaxis and the risk of ACM**

**Supplementary Figure 13. Forest plot: amiodarone prophylaxis and the risk of heart block**

**Supplementary Figure 14. Forest plot: amiodarone prophylaxis and the risk of hypotension**

**
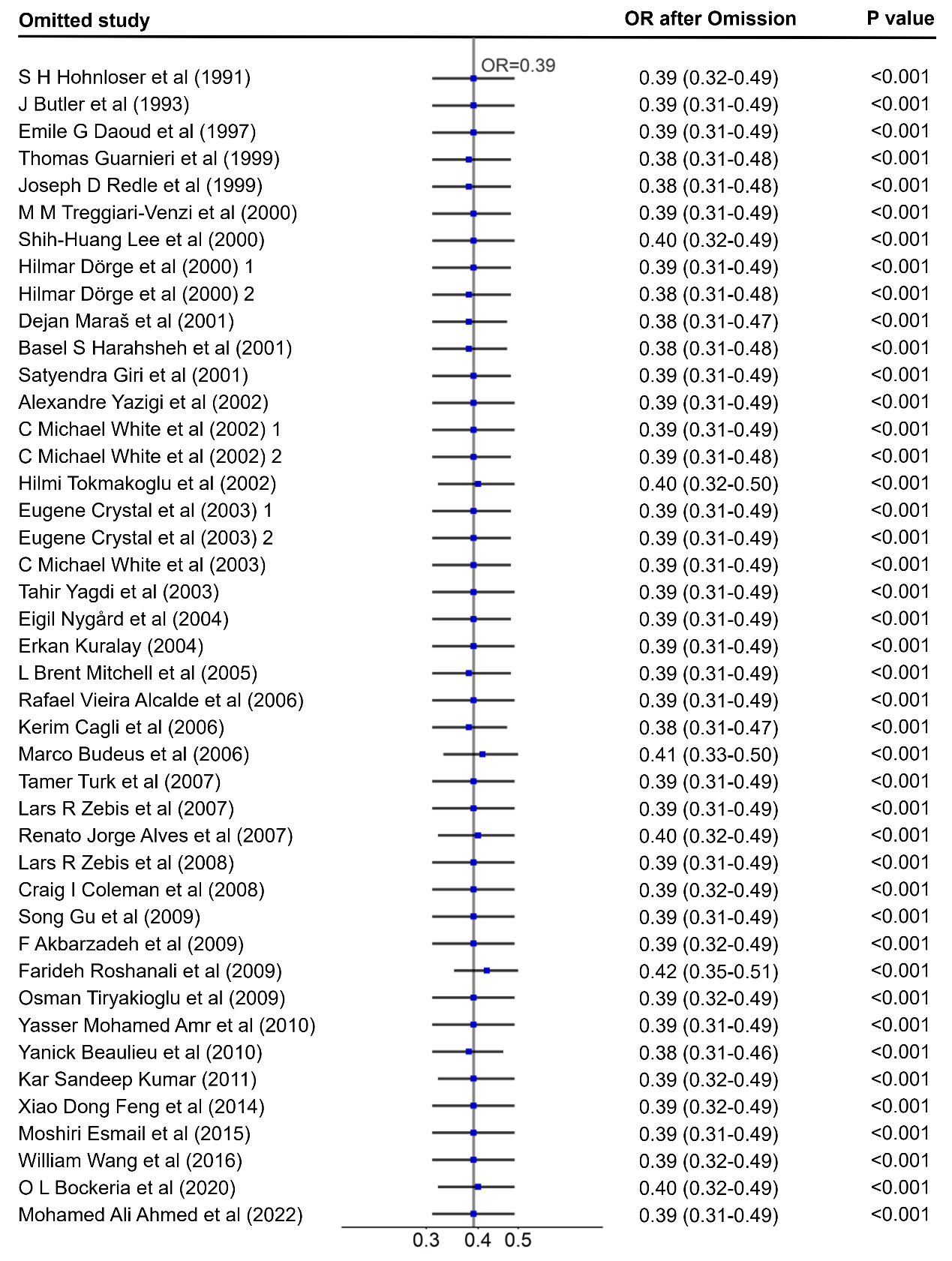
**

**Supplementary Figure 1. Forest plot: leave-one-out method**

**
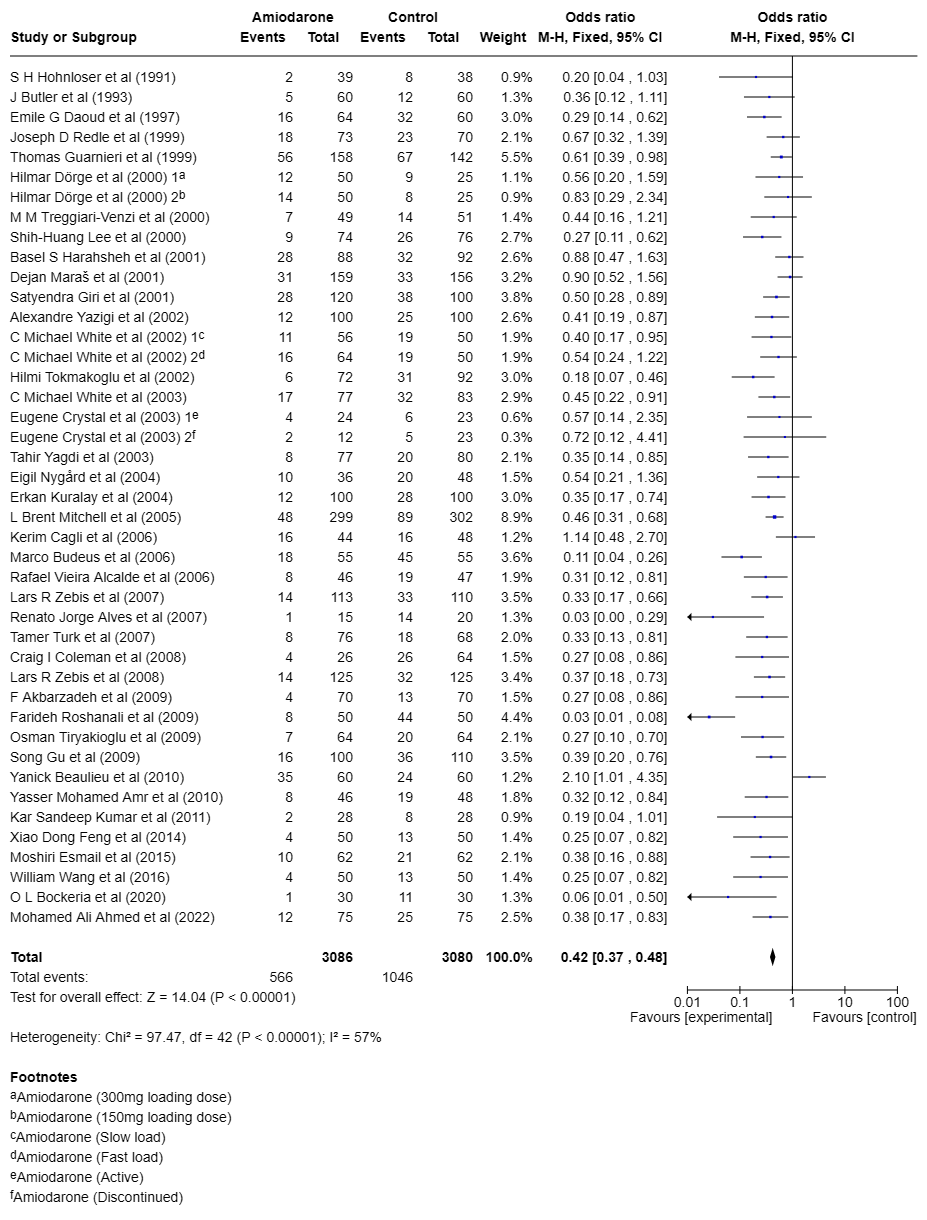
**

**Supplementary Figure 2. Forest plot:** **fixed-effect model**

**
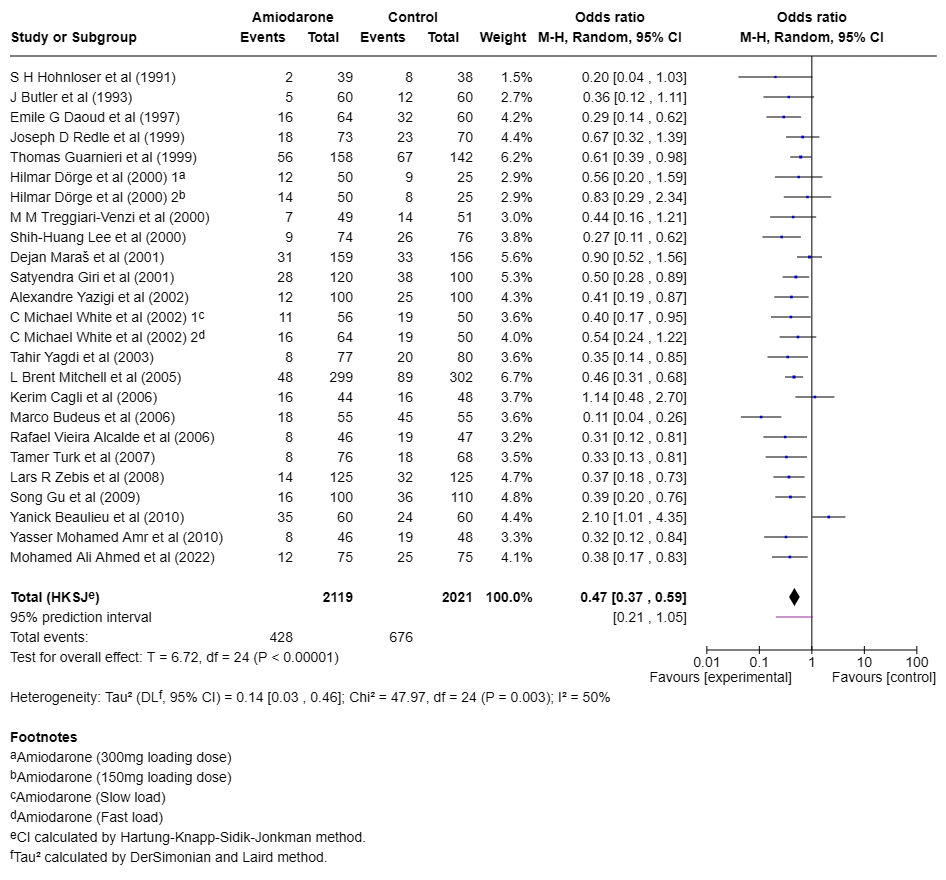
**

**Supplementary Figure 3. Forest plot: studies with low to moderate risk of bias**

**
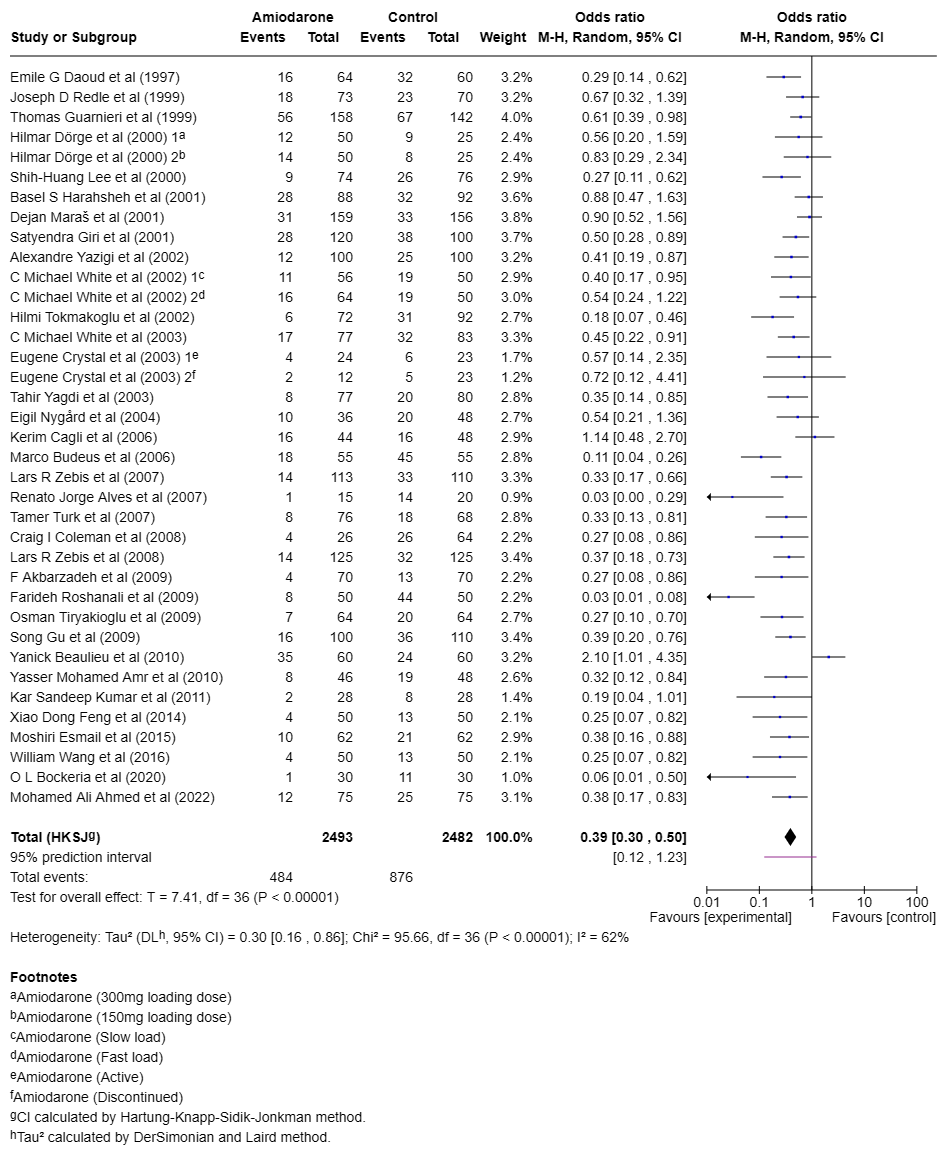
**

**Supplementary Figure 4. Forest plot: studies reporting POAF as the sole outcome**

**
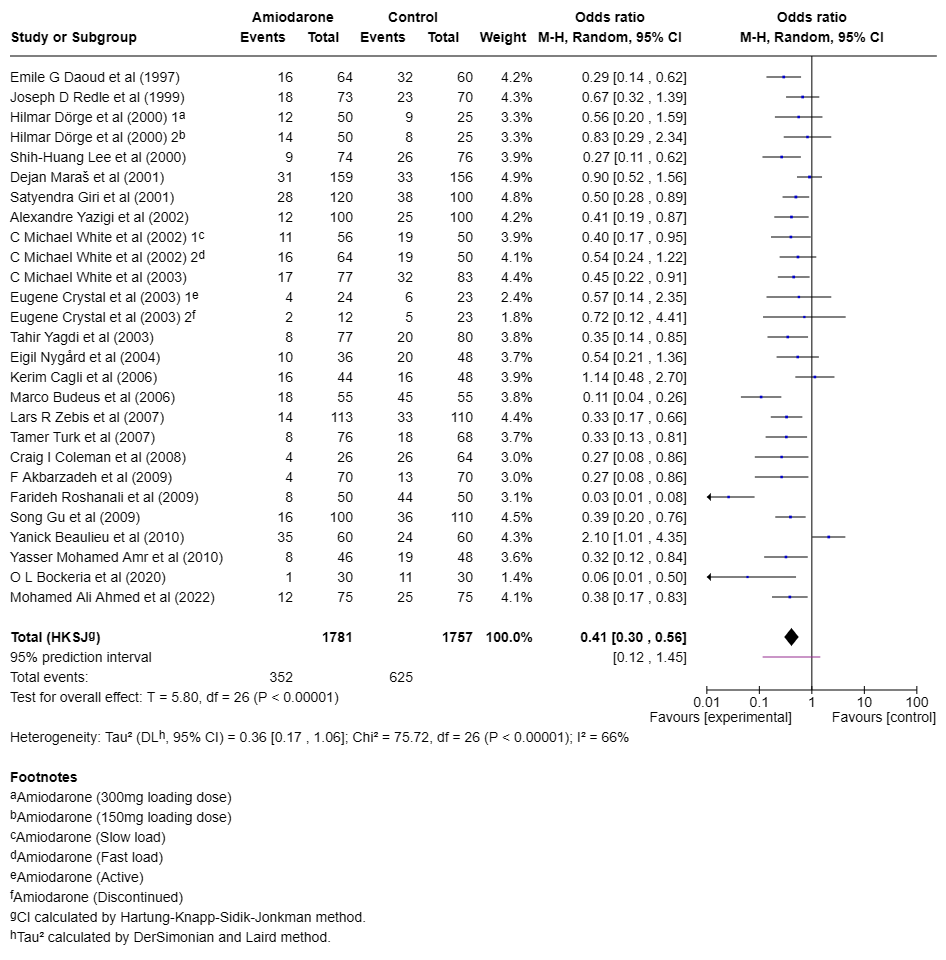
**

**Supplementary Figure 5. Forest plot: studies with a complete POAF definition**

**
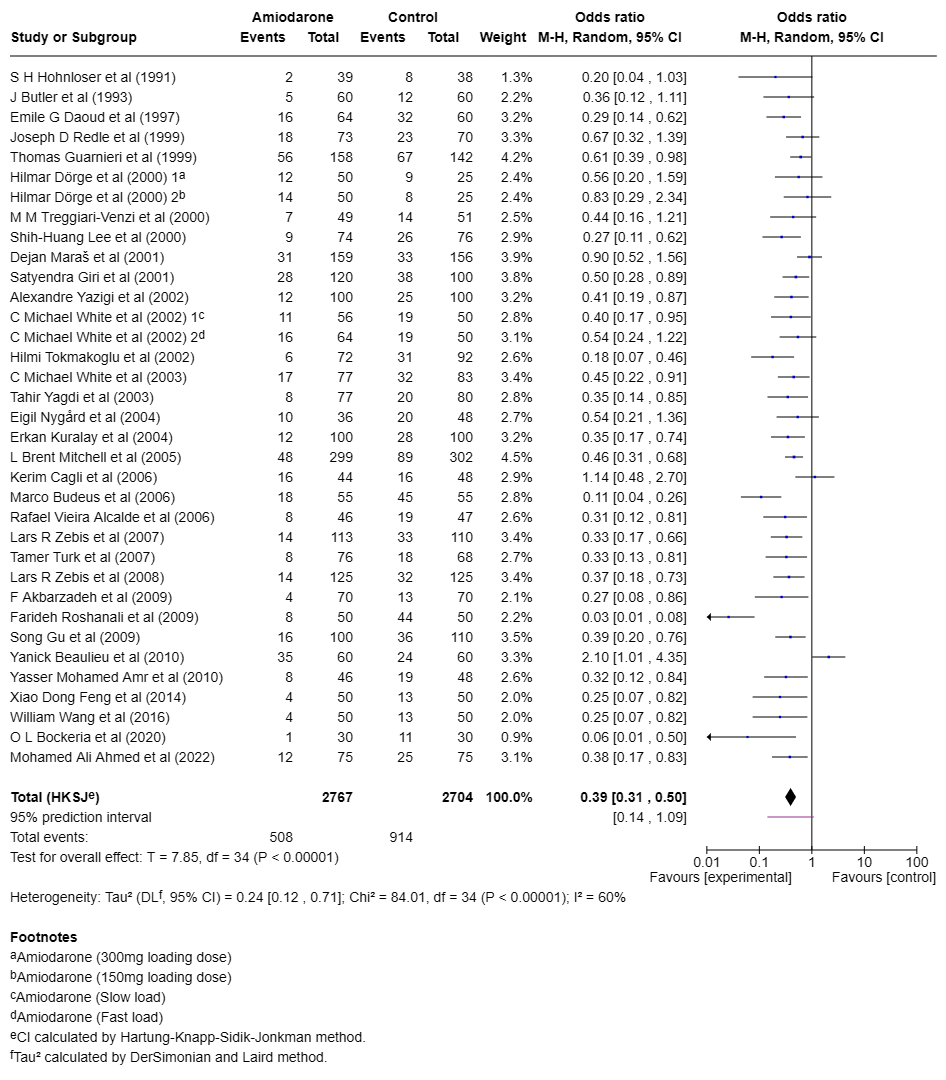
**

**Supplementary Figure 6. Forest plot: studies with continuous ECG monitoring**


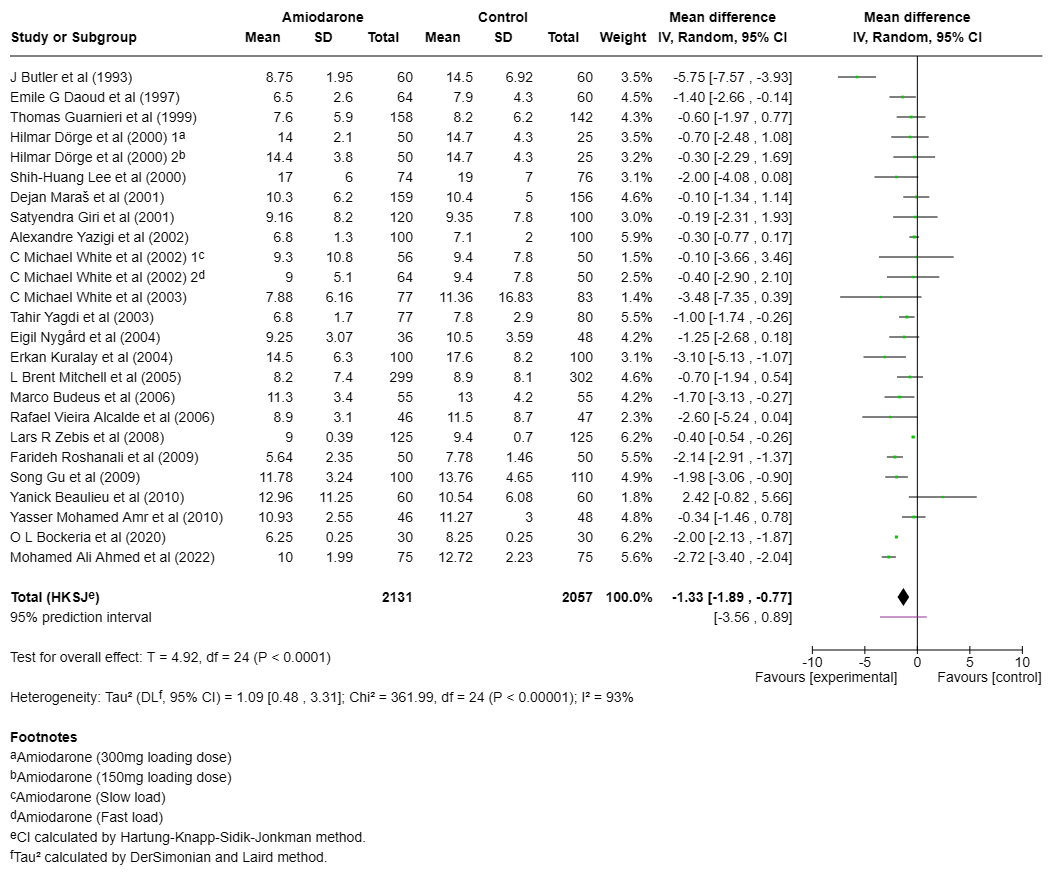


**Supplementary Figure 7. Forest plot: amiodarone prophylaxis and HLOS**

**
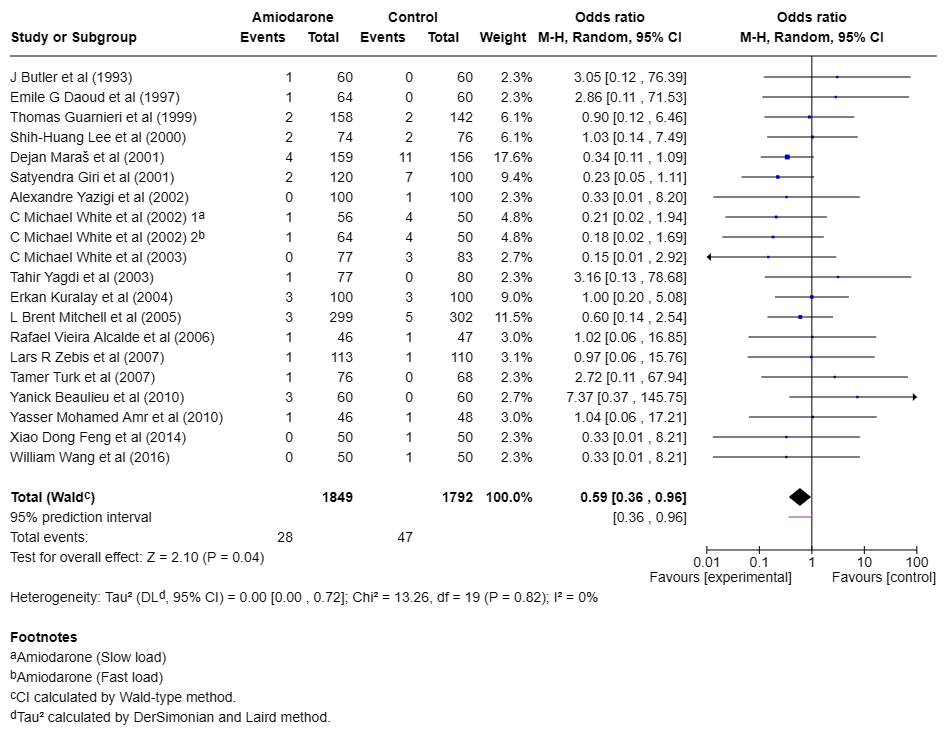
**

**Supplementary Figure 8. Forest plot: amiodarone prophylaxis and the risk of CVA**

**
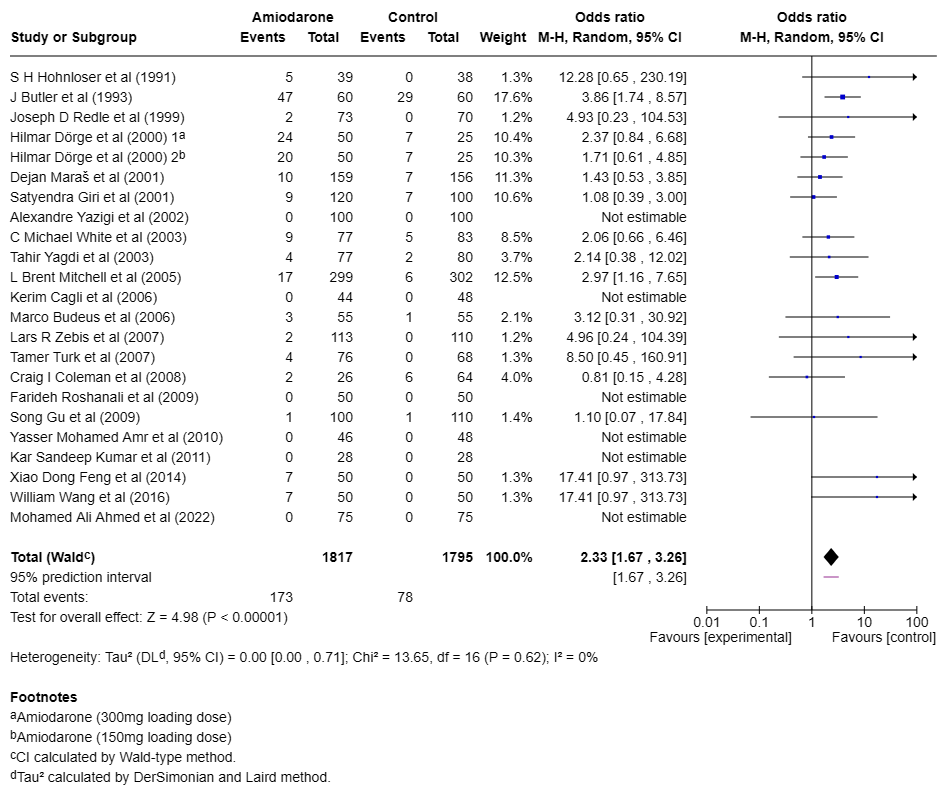
**

**Supplementary Figure 9. Forest plot: amiodarone prophylaxis and the risk of bradycardia**

**
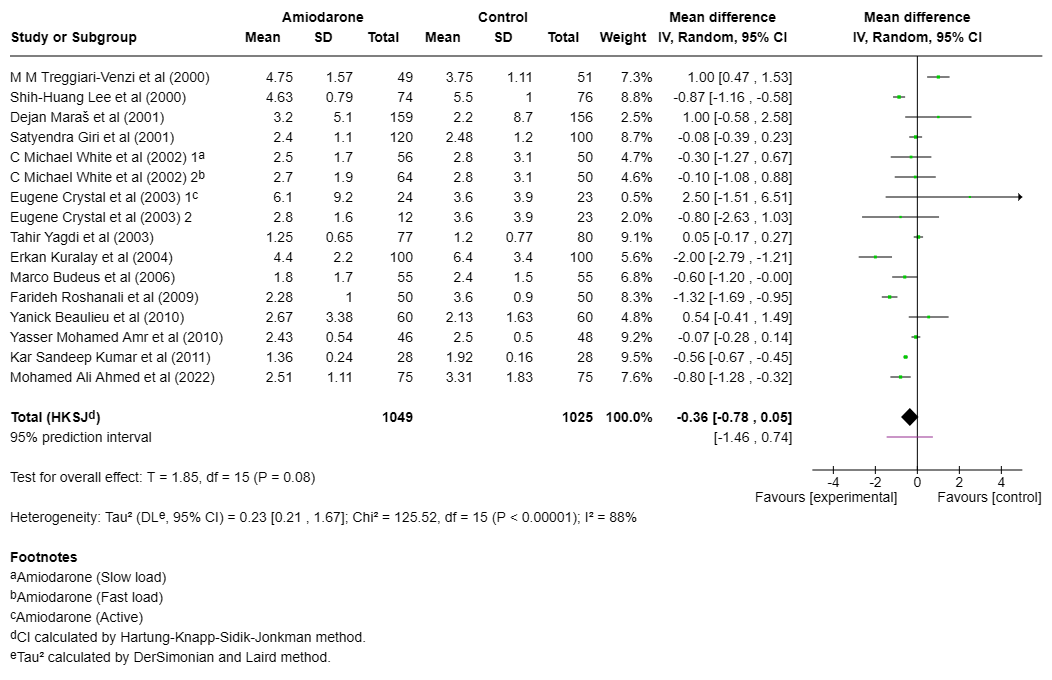
**

**Supplementary Figure 10. Forest plot: amiodarone prophylaxis and ILOS**

**
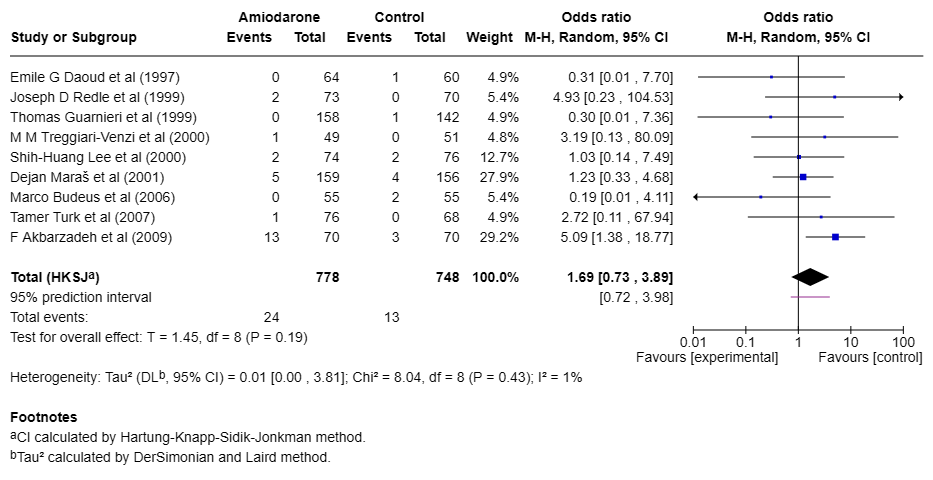
**

**Supplementary Figure 11. Forest plot: amiodarone prophylaxis and the risk of CVM**

**
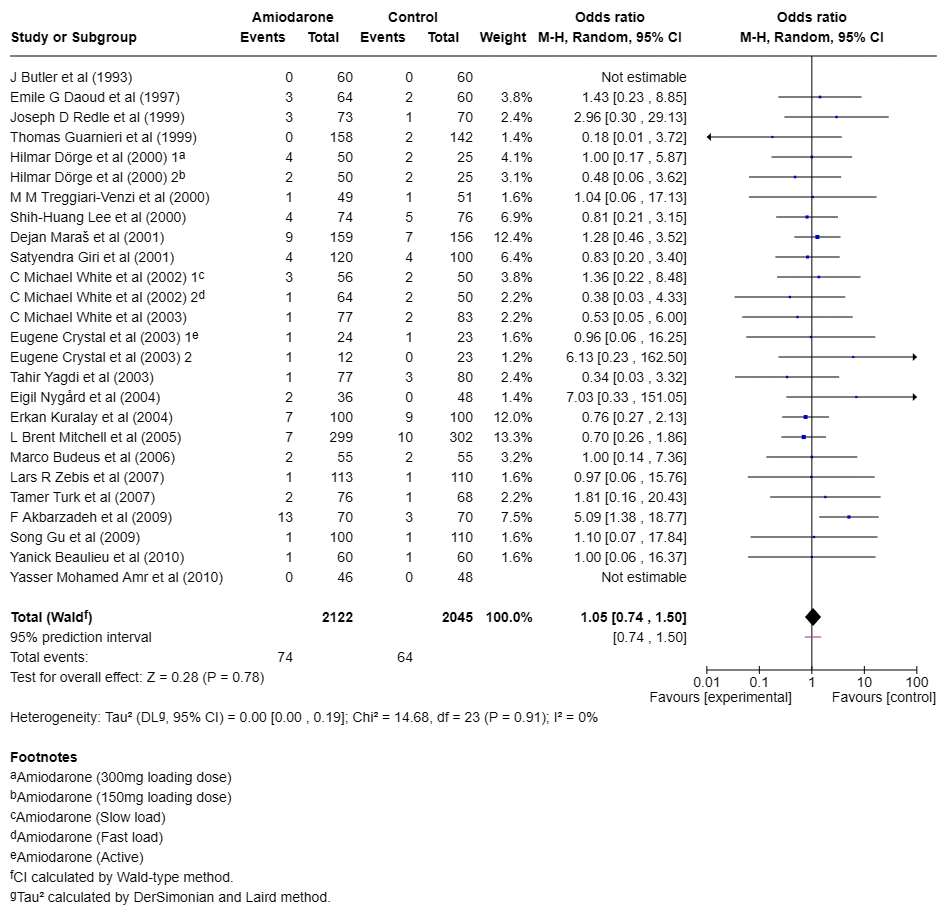
**

**Supplementary Figure 12. Forest plot: amiodarone prophylaxis and the risk of ACM**

**
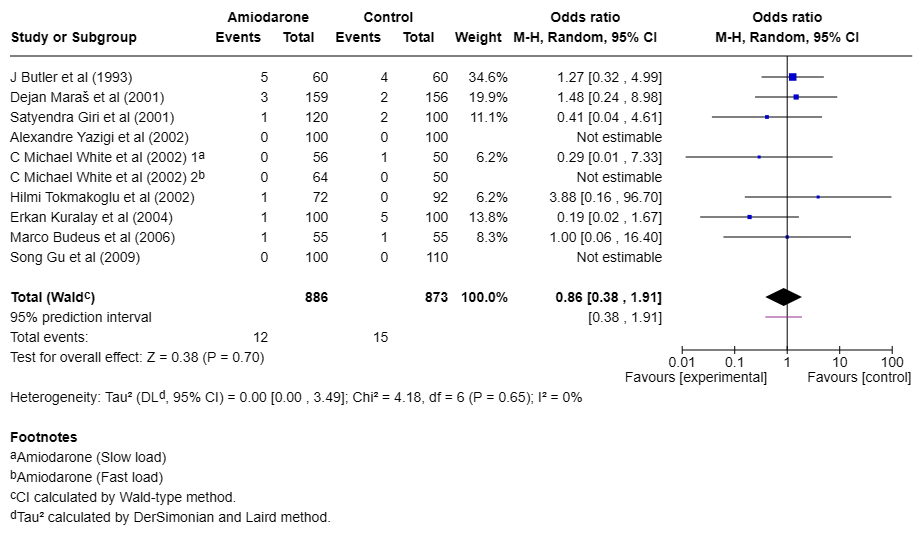
**

**Supplementary Figure 13. Forest plot: amiodarone prophylaxis and the risk of heart block**

**
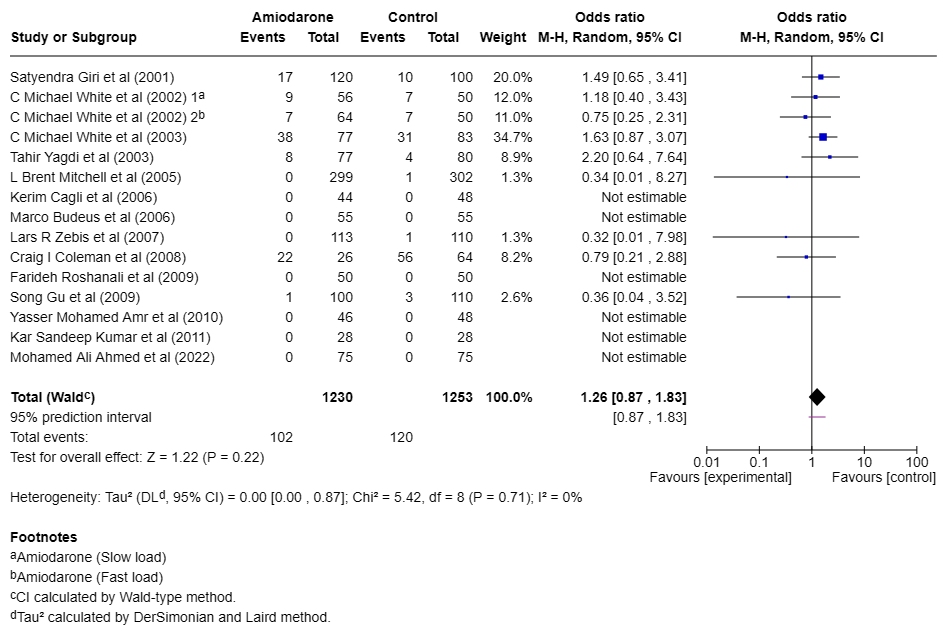
**

**Supplementary Figure 14. Forest plot: amiodarone prophylaxis and the risk of hypotension**
