# Supplementary material for: Meta-analysis of perioperative amiodarone for prevention of postoperative atrial fibrillation (POAF) in cardiac surgery patients: update and reevaluation of timing, route, and dosage
Source: BMC Cardiovasc Disord. 2026 May 7;26:547. doi: 10.1186/s12872-026-05813-w (PMC13321565; doi:10.1186/s12872-026-05813-w)
Supplement: Supplementary file 3 — Supplementary Material 3. [file 12872_2026_5813_MOESM3_ESM.docx]

| **Meta Analysis of Perioperative Amiodarone for Prevention of Postoperative Atrial Fibrillation (POAF) in Cardiac Surgery Patients: Update and Reevaluation of Route, Timing, and Dosage**  **Bibliography:** | | | | | | | | | | | |
| --- | --- | --- | --- | --- | --- | --- | --- | --- | --- | --- | --- |
| **Certainty assessment** | | | | | | | **Summary of findings** | | | | |
| **Participants (studies) Follow-up** | **Risk of bias** | **Inconsistency** | **Indirectness** | **Imprecision** | **Publication bias** | **Overall certainty of evidence** | **Study event rates (%)** | | **Relative effect (95% CI)** | **Anticipated absolute effects** | |
|  |  |  |  |  |  |  | **With placebo or no-treatment** | **With Amiodarone** |  | **Risk with placebo or no-treatment** | **Risk difference with Amiodarone** |
| **Postoperative atrial fibrillation** | | | | | | | | | | | |
| 6166 (40 RCTs) | serious^a^ | serious^b^ | not serious^c^ | not serious^d^ | publication bias strongly suspected^e,f,g^ | ⨁◯◯◯ Very low^a,b,c,d,e,f,g^ | 1046/3080 (34.0%) | 566/3086 (18.3%) | **OR 0.39** (0.31 to 0.49) | 1046/3080 (34.0%) | **173 fewer per 1,000** (from 202 fewer to 138 fewer) |

**CI:** confidence interval; **OR:** odds ratio

#### Explanations

a. Of the 40 studies included in this analysis, 8 (20%) were judged as low risk, 15 (37.5%) raised some concerns, and 17 (42.5%) were deemed high risk of bias. The pooled analysis of all 40 trials demonstrated that amiodarone significantly reduced the risk of postoperative atrial fibrillation (OR = 0.39, 95% CI: 0.31–0.49, P < 0.00001).

b. There is significant heterogeneity: Tau² (DLh, 95% CI) = 0.23 [0.12 , 0.66]; Chi² = 97.47, df = 42 (P < 0.00001); I² = 57%

c. No significant indirectness was identified in the evidence. All included studies directly addressed the clinical question of this systematic review in terms of population, interventions, comparisons, and outcomes (PICO). The population consisted exclusively of cardiac surgery patients. The intervention was amiodarone in all studies, compared against either placebo or standard care. The primary outcome was consistently postoperative atrial fibrillation across all studies.

d. No concerns regarding imprecision were identified. This conclusion is supported by a large number of patients and events, with a narrow confidence interval for the effect estimate that lies entirely within the range indicating significant benefit. The pooled analysis of 40 trials including 6,166 patients and 1,614 total events demonstrated a highly significant reduction in POAF (OR = 0.39, 95% CI: 0.31–0.49, P < 0.00001), with both upper (0.49) and lower (0.31) limits representing a clear and clinically meaningful risk reduction.

e. The plot exhibited some asymmetry, with data points indicative of extreme treatment benefits. Egger's test (t = -3.398, P = 0.002), Harbord's test (t = -2.064, P = 0.045), and Peters' test (t = -2.236, P = 0.031) indicated the potential for publication bias.

f. We assessed the possibility of upgrading the quality of evidence due to a large effect size. Although the risk ratio (RR) was 0.52, this value did not meet the GRADE threshold for a 'large effect' (RR < 0.5). Consequently, the evidence level was not upgraded.

g. According to GRADE guidance, this specific upgrade criterion is not applicable to a body of evidence derived from randomized controlled trials and was therefore not applied.

| **Meta Analysis of Perioperative Amiodarone for Prevention of Postoperative Atrial Fibrillation (POAF) in Cardiac Surgery Patients: Update and Reevaluation of Route, Timing, and Dosage**  **Bibliography:** | | | | | | | | | | | |
| --- | --- | --- | --- | --- | --- | --- | --- | --- | --- | --- | --- |
| **Certainty assessment** | | | | | | | **Summary of findings** | | | | |
| **Participants (studies) Follow-up** | **Risk of bias** | **Inconsistency** | **Indirectness** | **Imprecision** | **Publication bias** | **Overall certainty of evidence** | **Study event rates (%)** | | **Relative effect (95% CI)** | **Anticipated absolute effects** | |
|  |  |  |  |  |  |  | **With placebo or no-treatment** | **With Amiodarone** |  | **Risk with placebo or no-treatment** | **Risk difference with Amiodarone** |
| **Postoperative Atrial Fibrillation** | | | | | | | | | | | |
| 4140 (23 RCTs) | not serious^a^ | serious^b^ | not serious^c^ | not serious^d^ | none^e,f,g^ | ⨁⨁⨁◯ Moderate^a,b,c,d,e,f,g^ | 428/2021 (21.2%) | 676/2119 (31.9%) | **OR 0.47** (0.37 to 0.59) | 428/2021 (21.2%) | **100 fewer per 1,000** (from 121 fewer to 75 fewer) |

**CI:** confidence interval; **OR:** odds ratio

#### Explanations

a. Only 23 studies assessed as "low risk" and "some concerns" by the ROB2 tool were included here.The pooled analysis of 23 trials demonstrated that amiodarone significantly reduced the risk of postoperative atrial fibrillation (OR = 0.47, 95% CI: 0.37–0.59, P < 0.00001).

b. There is significant heterogeneity: Tau² (DLf, 95% CI) = 0.14 [0.03 , 0.46]; Chi² = 47.97, df = 24 (P = 0.003); I² = 50%.

c. No significant indirectness was identified in the evidence. All included studies directly addressed the clinical question of this systematic review in terms of population, interventions, comparisons, and outcomes (PICO). The population consisted exclusively of cardiac surgery patients. The intervention was amiodarone in all studies, compared against either placebo or standard care. The primary outcome was consistently postoperative atrial fibrillation across all studies.

d. No concerns regarding imprecision were identified. This conclusion is supported by a large number of patients and events, with a narrow confidence interval for the effect estimate that lies entirely within the range indicating significant benefit. The pooled analysis of 23 trials including 2,140 patients and 428 total events demonstrated a highly significant reduction in POAF (OR = 0.47, 95% CI: 0.37–0.59, P < 0.00001), with both upper (0.59) and lower (0.37) limits representing a clear and clinically meaningful risk reduction.

e. The plot did not exhibit asymmetry. Egger's test (t = -1.343, P = 0.192), Harbord's test (t = -0.835, P = 0.412), and Peters' test (t = -0.472, P = 0.641) indicated may not be publication bias.

f. We assessed the possibility of upgrading the quality of evidence due to a large effect size. Although the risk ratio (RR) was 0.59, this value did not meet the GRADE threshold for a 'large effect' (RR < 0.5). Consequently, the evidence level was not upgraded.

g. According to GRADE guidance, this specific upgrade criterion is not applicable to a body of evidence derived from randomized controlled trials and was therefore not applied.
